# Molecular architecture and activation of the insecticidal protein Vip3Aa from *Bacillus thuringiensis*

**DOI:** 10.1038/s41467-020-17758-5

**Published:** 2020-08-07

**Authors:** Rafael Núñez-Ramírez, Juanjo Huesa, Yolanda Bel, Juan Ferré, Patricia Casino, Ernesto Arias-Palomo

**Affiliations:** 1 Centro de Investigaciones Biológicas Margarita Salas, CSIC, 28040 Madrid, Spain; 2 Departament de Bioquímica i Biologia Molecular, Universitat de València, Dr. Moliner 50, 46100 Burjassot, Spain; 3 Estructura de Recerca Interdisciplinar en Biotecnologia i Biomedicina (ERI BIOTECMED), Universitat de València, Dr. Moliner 50, 46100 Burjassot, Spain; 4 Department of Genetics, Universitat de València, Dr. Moliner 50, 46100 Burjassot, Spain; 5 CIBER de Enfermedades Raras (CIBERER-ISCIII), Madrid, Spain

**Keywords:** Molecular biology, Structural biology, Electron microscopy

## Abstract

*Bacillus thuringiensis* Vip3 (Vegetative Insecticidal Protein 3) toxins are widely used in biotech crops to control Lepidopteran pests. These proteins are produced as inactive protoxins that need to be activated by midgut proteases to trigger cell death. However, little is known about their three-dimensional organization and activation mechanism at the molecular level. Here, we have determined the structures of the protoxin and the protease-activated state of Vip3Aa at 2.9 Å using cryo-electron microscopy. The reconstructions show that the protoxin assembles into a pyramid-shaped tetramer with the C-terminal domains exposed to the solvent and the N-terminal region folded into a spring-loaded apex that, after protease activation, drastically remodels into an extended needle by a mechanism akin to that of influenza haemagglutinin. These results provide the molecular basis for Vip3 activation and function, and serves as a strong foundation for the development of more efficient insecticidal proteins.

## Abstract (web summary)

Bacillus thuringiensis Vip3 (Vegetative Insecticidal Protein 3) toxins are widely used in biotech crops to control Lepidopteran pests. Here authors present the cryo-EM structures of the protoxin and the protease-activated state of Vip3Aa which shed light on the molecular basis for Vip3 activation.

## Introduction

The bacterium *Bacillus thuringiensis* (Bt) synthetizes potent insecticidal proteins, such as the Cry toxins generated during sporulation, commonly used in agriculture^1,2^. In addition, Bt secretes to the medium numerous vegetative insecticidal proteins (Vip) that have been classified into three different families^3^. Notably, members of the Vip3 family have shown to have a broad-spectrum activity against Lepidoptera^4^ and, since they target binding sites different to those of Cry proteins in the insect membranes^5–11^, they are considered a good complement to the action of these toxins. Crystal/spore mixtures of some Bt strains are indeed the active ingredient of bioinsecticides used in traditional and organic farming, and the genes coding for Cry and Vip3 proteins have been introduced in plants (Bt crops) to protect them from insect pests^2,3^.

Vip3 proteins are synthesized in an inactive state, as protoxins, and they require to be activated by proteases present in the midgut tract of the insect. Trypsin-like enzymes cleave the molecule at residue 198, generating two fragments of around 19 and 65 kDa that remain strongly associated^7,12–15^. The activated protein then recognizes specific receptors in the brush border of columnar cells, whose identity is still under debate, and it has been proposed that binding to some of them can induce internalization^16–18^. Notably, although several cell lethality pathways have been proposed, numerous studies indicate that Vip3 proteins form pores in the membrane to trigger cell death^6,8,13,19,20^.

The first member of the Vip3 family was discovered more than 20 years ago. However, numerous questions persist regarding the three-dimensional organization, activation and mode of action of these proteins at the molecular level. To shed light on some of these questions, we have determined the near-atomic resolution structures of Vip3Aa in the protoxin and activated states by cryo-electron microscopy (cryo-EM). The reconstruction of the uncleaved protein reveals that it assembles into a highly stable tetrameric dimer of dimers that contains three putative sugar-binding domains poised for receptor engagement. The trypsin-activated structure unravels the large conformational changes that occur upon protease digestion in the N-terminal region, and that leads to the formation of an extended four-helix coiled coil. Collectively, our work provides a high-resolution view of a Vip3 member in the protoxin and toxin conformation and allows us to propose an activation mechanism for this family of entomopathogenic proteins that both parallels and diverges from other toxins and viral fusion proteins.

## Results

### Molecular architecture of the Vip3Aa protoxin

**Fig. 1:**
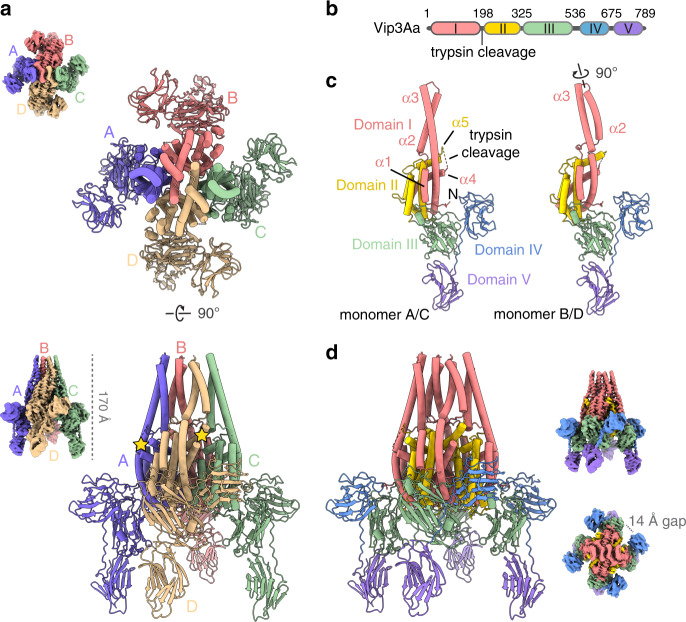
Protoxin structure of Vip3Aa from *Bacillus thuringiensis*. **a** Two orthogonal views of the Vip3Aa protoxin. Each monomer has been colored differently. Yellow stars mark the position of the trypsin cleavage site in two of the monomers. Unsharpened EM map shown on the left. **b** Primary structure of Vip3Aa. **c** Vip3Aa is formed by two types of monomers, outer (left), and inner (right), that are mainly differentiated by a ~90° rotation of the α2–α3 helical bundle. **d** Vip3Aa tetramer colored according to the domain organization. Two orthogonal views of the EM reconstruction are shown on the right.

To better understand the three-dimensional organization and activation mechanism of these toxins, purified Vip3Aa was applied to electron microscopy grids coated with a thin carbon layer and analyzed using cryo-EM. The reference-free 2D averages of the motion-corrected micrographs show, in agreement with previous reports, the tetrameric organization of the protoxin^12,15,21–24^ (Fig. 1, Supplementary Figs. S1,  S2). Interestingly, the three-dimensional reconstruction, which converged at a global resolution of 2.9 Å (Fig. 1a, Supplementary Fig. S1 and Supplementary Video 1), revealed that the Vip3 tetramer assembles into a pyramid-shaped structure where the N-terminal region forms the core and apex, and the C-terminal domains are exposed to the solvent. Overall, the high-resolution structural features and the quality of the EM map have permitted unambiguous de novo atomic modeling of the Vip3Aa protoxin (Fig. 1a and Supplementary Table S1).

The cryo-EM structure of the protoxin reveals key insights into the molecular architecture of the protein, and shows that Vip3 is composed of five distinct domains (Fig. 1b–d and Supplementary Fig. S3), confirming earlier predictions^23^. Domain I extends from the N-terminus to the primary protease cleavage site (K198)^14,25^, and contains four long, highly curved α-helices (α1–α4). The first helix is markedly amphipathic (Supplementary Fig. S4), and the second and third helices form an antiparallel bundle that adopts a different configuration in the outer (chains A and C) and inner monomers (chains B and D) (Fig. 1c). In particular, the variable length of the α1–α2 loop allows the second and third helices of the internal monomers to rotate ∼90° compared with the external ones (Supplementary Video 2). This rotation further increases the curvature and tension of this element and leads to the formation of an eight-helix bundle that protrudes as a stalk from the tetrameric core formed by the subsequent domain (Fig. 1a, d). Domain II (residues 200–325) starts immediately after the cleavage site, which is located in a fully exposed loop that connects helix α4 and α5 (Fig. 1c). Although some of the most solvent accessible residues (T190-P202) were flexible—probably to facilitate trypsin cleavage—and were not modeled, there is clear density for the entire loop in the unsharpened EM map (Supplementary Fig. S5a, b). Notably, this second domain, composed of five α helices, embodies the core of the tetramer (Fig. 1d) and plays a structural role mainly through two extended loops (comprising residues 221–226 and 239–247, respectively) that project toward the adjacent subunit and stabilize the Vip3 oligomer (Supplementary Fig. S5c, d).

**Fig. 2:**
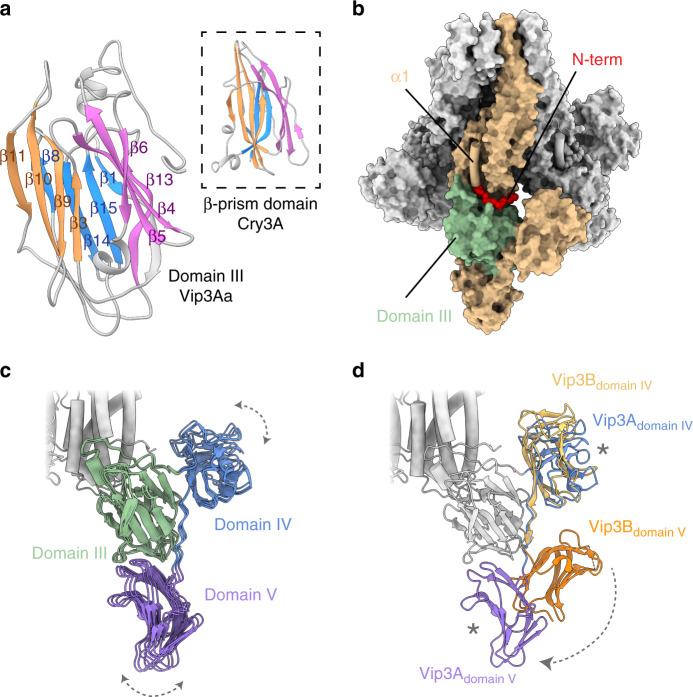
The flexible C-terminal domains contain motifs found in other toxins. **a** Domain III forms a β-prism fold similar to that found in the Cry toxins. **b** The N-terminal region of the four monomers (red) is inserted into a groove formed between domain III (green) and the core of the particle. **c** Superposition of domains III, IV and V, docked into the three most distant positions found in the focused 3D classification, shows the flexibility of these domains in the Vip3Aa reconstruction. **d** Although the comparison with the Vip3B protoxin (PDB ID 6V1V) reveals a similar overall architecture, the orientation of domain V is markedly different to that found in Vip3Aa. The putative sugar binding site at domain IV and V is labeled as an asterisk.

In sharp contrast, the C-terminal region of Vip3 is composed of three globular domains that are mostly solvent exposed and do not show any interactions between different monomers. Indeed, there is a clear gap of ~14 Å (minimum distance) that separates these domains from the neighboring monomer (Fig. 1c, d). Domain III comprises residues 328–532 and contains three antiparallel β-sheets that form a β-prism fold strikingly similar to that found in members of the Cry insecticidal δ-endotoxins^26^ (Fig. 2a). Although in some Cry toxins this motif has been shown to recognize the sugar moieties that decorate the receptors of the target cell^27,28^, the exact role in Vip3A proteins has not yet been fully elucidated. This third domain, interestingly, is interacting and clamping the most N-terminal segment of the protein (residues 14–23), against the core of the tetramer (Fig. 2b).

The EM density for the last two domains is less well ordered. However, we were able to improve significantly the quality of the density in this region by performing a cycle of symmetry expansion, particle subtraction and focussed classification. The analysis confirmed the inherent flexibility of these domains—that make loose contacts with domain III—and allowed us to determine their 3D structure (Figs. 1, 2c and Supplementary Video 3). Notably, these two domains bear similar CBM (carbohydrate-binding module) folds that are connected by a long linker. Although the precise type of substrates that these domains could bind was not clear, a recent study indicates that they have a strong preference for chitosan and chitin^29^, and the DALI server revealed that they show similarity to glycoside hydrolases, which are often found in tandem to increase affinity for polysaccharides^30,31^.

Interestingly, during the preparation of this manuscript the protoxin structure of Vip3B was published^32^, which allows us to compare the molecular architecture of two different inactive Vip3 variants. The structural analysis reveals that both proteins share a similar overall tetrameric organization. More precisely, domains I, II, and III show a remarkable structural similarity (RMSD value of ∼1 Å for 1932 residues), consistent with their elevated sequence homology (∼73%) (Supplementary Fig. S6). The isolated structures of the last two domains are also analogous. However, their position and orientation with respect to the core of the molecule differs slightly for domain IV and more pronouncedly for domain V, which rotates ~100° (Fig. 2d and Supplementary Fig. S6). These results further confirm the flexible nature of the CBM domains that, importantly, maintain the putative glycan binding sites exposed to the solvent.

### Trypsin activation triggers a large molecular reorganization

**Fig. 3:**
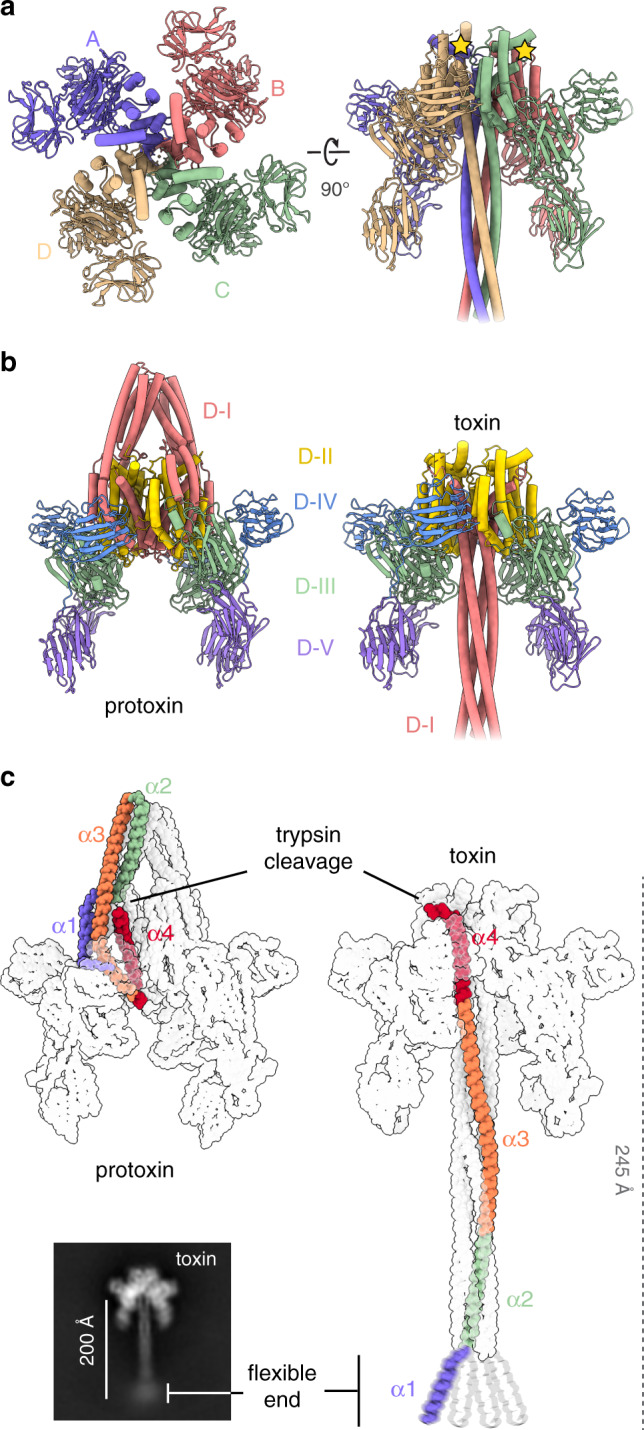
Molecular architecture of the trypsin activated Vip3Aa toxin. **a** Two orthogonal views of the Vip3Aa toxin show that the four monomers are organized around a central pore. The different monomers have been colored as in Fig. 1a. Yellow stars indicate the position of the trypsin cleavage site in two of the monomers. **b** Similar views of the Vip3Aa protoxin and toxin colored by domain organization as in Fig. 1b. Domains are labeled as D-I through D-V **c** The alpha helices that comprise domain I undergo a large reorganization that leads to the formation of an extended coiled coil. The four helical bundle is ordered starting from residue ~45, and reference-free 2D averages show that the most N-terminal region appears to be flexible.

The analysis of the protoxin structure did not initially explain how protease digestion triggers the activation of Vip3 that leads to cell death. We therefore decided to solve the three-dimensional structure of Vip3Aa after trypsin treatment. The purified protein was first incubated with the protease using conditions that have been reported to produce full activation^3,14^ (Supplementary Fig. S7a). The sample was then deposited onto open-hole grids coated with a thin layer of homemade carbon, flash-frozen, and examined using cryo-EM. Interestingly, although the efficiency of the digestion was elevated (>95% according the PAGE gel), the 2D class averages showed that ~30% of the molecules maintained a configuration remarkably similar to that of the undigested sample (Supplementary Fig. S7b). This observation probably reflects that, although protease cleavage is required, other factors might modulate the activation of Vip3. Notably, however, the majority (~70%) of the particles adopted a syringe-like structure -toxin from now on- that after image processing converged into a 2.9 Å reconstruction (Fig. 3, Supplementary Figs. S7,  S8).

Inspection of the structure of the protease-activated toxin reveals a tetramer where the four monomers adopt an identical conformation arranged around the fourfold symmetry axis of the particle, and confirms that the cleaved fragments remain tightly associated^7,12,14,15^ (Fig. 3a, b and Supplementary Video 4). Activated Vip3Aa shows the same domain organization described in the protoxin state, and domains II–V maintain a similar global architecture (Fig. 3b and Supplementary Fig. S9). Domain I, however, suffers a large conformational change that remodels the apex of the pyramid into a ~200 Å-long needle. Notably, the required set of movements can proceed unimpeded though the gap located between monomers. In particular, three antiparallel N-terminal helices (α2–α4) undergo a set of rotations, in a manner akin to a folding cane, to form a long continuous helix that assembles into a parallel four-helical coiled-coil in the digested Vip3Aa tetramer (Fig. 3c). The cryo-EM analysis shows that the coiled-coil is relatively ordered starting at the beginning of α2 (~D45), and that the flexible end is formed by the most N-terminal region of the protein, which contain the residues forming the first alpha helix in the protoxin state (Fig. 3c).

**Fig. 4:**
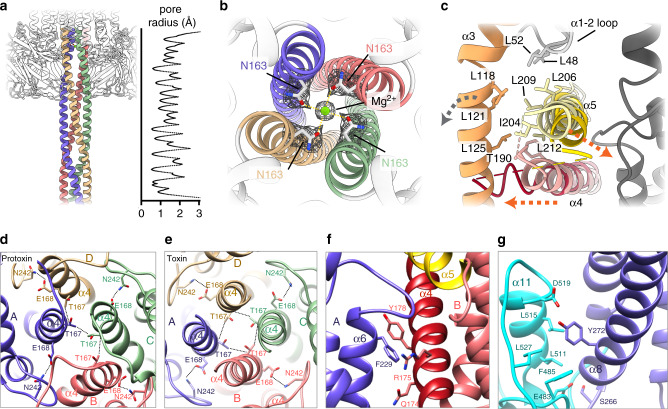
Dimensions and formation of the coiled coil. **a** Side view of the coiled coil region modeled at atomic resolution (residues 95–167). The corresponding pore radius is plotted on the right. Source data are provided as a Source Data file. **b** A magnesium ion, coordinated by Asn 163, was modeled into the central pore of the cryo-EM structure. **c** A hydrophobic interaction network, formed by residues located in α3, α4, α5, and the α1–α2 loop (light orange, red, yellow and gray respectively), helps to maintain Vip3Aa in an inactive configuration. Helices α4 and α5 reposition upon trypsin cleavage (dark red and yellow helices, orange arrows indicate the direction of the movement), disrupting the hydrophobic patch and promoting the conformational change of the N-terminal region of the protein (indicated by a gray arrow). **d** Representation of inter-monomer interactions between residue T167 (domain I), as well as the intra-monomer interactions between E168 (domain I) and N242 (domain II), in the protoxin. **e** Same contacts shown in panel **d** in the toxin structure. **f** Inter-monomer interactions between F229 at domain II with residues located at the central part of α4 in the adjacent monomer (dark red), highlighting α5 (in yellow). **g** Intra-monomer interactions between domain II (dark blue) and domain III (light blue).

Close examination of the structure of the toxin revealed that the coiled-coil of Vip3A, which resembles other four-helix bundles such as the SNARE complex^33,34^ (RMSD of 3.4 Å for 232 residues), extends ~160 Å and that the average internal radius of the modeled section was 1.46 Å (Fig. 4a). Classic coiled-coils contain a series of heptad repeats (*abcdefg*) where the residues that line the lumen (*a* and *d*) are usually hydrophobic to maintain the distinctive knobs-into-holes packing of this motif^35,36^. In the case of Vip3Aa, although most residues found at positions *a* and *d* were hydrophobic (~80%), a significant fraction were polar, with asparagine being the most abundant one. Interestingly, comparative analysis of different structures, like that of the Sendai virus P protein, have shown that hydrophilic residues found in these positions—known as N*@d* layers—can bind ions and water molecules in the central cavity of the tetrameric coiled-coil^37,38^. Although the resolution of the cryo-EM reconstruction does not allow visualization of water molecules, there is a clear peak of density coordinated by four symmetry related asparagines (N163) that, based on the buffer composition (5 mM MgCl_2_), was assigned to a magnesium ion (Fig. 4b).

### Remodeling and molecular basis for the activation of Vip3Aa

Detailed comparison of the protoxin and toxin structures reveals the dramatic reorganization that the protein undergoes upon protease digestion, but what triggers the conformational change? Part of the answer is that the cleavage of the trypsin loop allows the re-configuration of the adjacent helices (α4 and α5) (Fig. 4c). The N-terminal region of α5 moves toward the neighbor monomer and disrupts a hydrophobic interaction network, between helix α3 and the loop between α1 and α2, that had been previously shown to be critical for the protein^39^. The C-terminal part of α4, in turn, moves against α3, applying pressure to further facilitate the conformational change that leads to the formation of the N-terminal coiled-coil and the activation of the protein. Concomitantly, several residues in the N-terminal part of α4 remodel to nucleate the formation of the coiled-coil. In this way, T167, which is generating a network of inter-monomer interactions in the protoxin, is the first residue to realign and nucleate the apical section of the coiled-coil, with the side chains pointing toward its central axis (Fig. 4d, e). Similarly, N163 is also forming an inter-monomeric network of interactions in the protoxin but relocates right one helix turn below T167 at the coiled-coil to bind the divalent ion observed in the toxin configuration (Fig. 4b). Strikingly, although the N-terminal part of α4 swivels during the activation of the protein, the C-terminal portion remains firmly attached to the domain II (Fig. 4f), which acts as an anchor point and provides a stable framework during the conformational changes that lead to the activation of the protein.

## Discussion

In 1991 the first 3D structure of a Cry protein was reported^26^, providing key insights into their function and facilitating the improvement of their insecticidal properties. In contrast, structural studies of Vip3A have lagged behind limiting the understanding of their function. The results presented here solve many long-standing questions concerning the molecular organization and activation of the Vip3 family of toxins and help to explain and reconcile some difficult-to-interpret functional and biological data obtained during the last decades. The structures, for instance, shed light on the controversial role of the N-terminal 198 amino acids (domain I)^3^ and the puzzling observation that after trypsinization this fragment remains associated to the rest of the protein. Domain I is indeed retained after protease digestion and stays tightly bound to the core of the protein through interactions with domain II, forming an essential part of the active toxin. Moreover, the structures explain why the exchange of this first domain between Vip3 variants can produce stable chimeras^12,40^, since the replacement of this highly conserved domain probably has a minor effect on the overall structure of the protein, and it also clarifies why attempts to produce Vip3 mutants in the trypsin loop or completely lacking domain I have been unsuccessful or have rendered an inactive or unstable proteins^12,25,41^.

In addition, the cryo-EM structures also reveal why some mutants have a dramatic effect on the stability and activity of the toxin. For instance, recent use of the alanine scanning technique has shown a cluster of residues around the interface of domain I and II (from residue 167 to 272) whose replacement by Ala highly decreased the insecticidal activity of Vip3Af to *Spodoptera frugiperda*^39^. Residues E168A, F229A, Y272A, and E483 maintain fundamentally the same contacts in the protoxin and toxin structures and most likely contribute to the stabilization of the protein during the needle formation. In this way, residue E168 interacts with N242, found in the domain II of the same monomer, and contributes to stabilize both domains during remodeling (Fig. 4d, e). Similarly, F229, which is located at α6 of domain II, interacts with residues in the central part of α4 of the adjacent monomer generating a hydrophobic pocket (Fig. 4f) that seems to maintain α4 bound to the core of the tetramer upon needle formation. Residue Y272, located at α8 in domain II, is setting network contacts with residues from α11 at the C-terminal end of domain III of the same monomer that are also maintained in the toxin structure (Fig. 4g). E483 reinforces this interaction engaging the base of α8 to bridge the core of the tetramer and the β-prism domain (Fig. 4g). Taken together, our data show that numerous mutants known to decrease the insecticidal activity lay in conserved key residues that have a crucial role in maintaining the integrity of the tetramer.

**Fig. 5:**
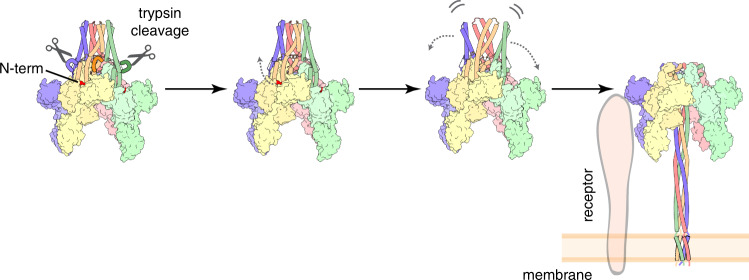
Activation mechanism of Vip3Aa. Protease digestion is required for Vip3 activation as it facilitates, possibly in conjunction with other factors, the remodeling of the spring-loaded helices that form the protoxin apex. This set of long-range conformational changes releases the N-terminus segment of the protein and leads to the formation of a four-helix coiled coil, which is sufficiently long to reach and permeate the lipid bilayer.

Overall, the structural transformations observed during Vip3 activation allow us to propose a spring-loaded mechanism that shares numerous similarities with that of influenza haemagglutinin and other viral fusion proteins^42,43^. It is well established that haemagglutinin is primed by protease digestion and that, after receptor engagement and stimulation by other factors, a hidden N-terminal insertion peptide unlocks from the core of the protein that undergoes a large conformational change to form a three-helix coiled-coil^42,44^. In the case of Vip3, the protein is first secreted and spontaneously assembles into a tetrameric protoxin. Following ingestion, the protein is processed by the insect’s midgut proteases and crosses the peritrophic membrane to interact with surrogate receptors in the apical membrane of the epithelial cells. Proteolytic cleavage is required to trigger a set of events that lead to the activation of the toxin. The N-terminus of the protein detaches from a pocket formed between domain III and the main body of the tetramer, a process that could be further stimulated by other factors like the interaction of the membrane receptors with the C-terminal region of Vip3, which could also increase the concentration of the toxin at the membrane (Fig. 5). Protease digestion, in addition, would release the accumulated tension in α3 and would allow the α2–α3 bundle to rotate and revert to the configuration found in the outer monomers (A and C), destabilizing the apex of the protoxin structure and promoting the formation of the tetrameric N-terminal coiled-coil.

Interestingly, it has been previously established that Vip3, similarly to the Cry toxins, forms pores in the insect cell and it is thought to act as an ion channel^6,8,13^. Notably, the coiled-coil observed upon trypsin digestion forms a 160 Å-long dipole that can accommodate small ions in the inner cavity and, therefore, the dimension of this four-helix needle would be sufficient to reach and pierce the lipid bilayer. It is not completely clear, however, whether the first α-helix that forms the flexible end of the toxin could aid in the process. Its length, commensurable with the width of the membrane^45^, and highly amphipathic nature suggests that it could become ordered in contact with the lipid bilayer to form a four-helix bundle similar to the Rocker transporter or the influenza M2 channel, which are known to be responsible of transporting protons and divalent metals through the membrane^46–49^. Further studies will be required to establish a detailed mechanism for receptor recognition and cell permeation.

In summary, this work offers a high-resolution picture of the architecture and activation mechanism of Vip3. The structural information and precise definition of the domain boundaries can help to generate chimeras with modified target specificity and, overall, these studies provide a molecular framework that can be used to guide future studies to develop engineered proteins with increased stability and insecticidal activity.

## Methods

### Protein expression and purification

The Vip3 protein used in this study was Vip3Aa16 (NCBI accession No. AAW65132), previously named Vip3LB^50^. The *vip3Aa16* gene (residues from 10 to 789) was cloned into the vector LIC 1.5 ampicillin resistant (pETNKI-StrepII3C-LIC-amp) containing an N-terminal StrepII-tag followed by a 3C PreScission protease cleavage site^51^ (the primers used were Vip3_start10+: CAGGGACCCGGTACAAGAGCCTTACCAAGTTTTATTG and Vip3-FL: CGAGGAGAAGCCCGGTTACTTAATAGAGACATCGTAAAAATGTAC). The protein was expressed in *Escherichia coli* C43 (DE3) cells grown in Luria-Bertani broth adding 0.5 mM isopropylthiogalactoside, upon reaching exponential phase, followed by incubation at 37 °C for 3 h. Cells were harvested by centrifugation at 4000 *g* for 15 min and stored at −20 °C until use. For protein purification, thawed cells were resuspended in buffer A (50 mM Tris-HCl pH 8.0, 500 mM NaCl, 50 mM MgCl_2_ plus 1 mM PMSF and 1 mM TCEP) and sonicated for 5 min. Then, the cell lysate was centrifuged at 25000 g for 30 min and the clarified supernatant was loaded into a 5 ml Streptrap column (GE, Healthcare). The protein was eluted in 2.5 mM *d*-Desthiobiotin dissolved in buffer A. The StrepII-tag was removed upon incubation with 3C PreScission protease, fused to an N-terminal GST-tag, at 4 °C overnight during a dialysis procedure to remove *d*-Desthiobiotin. Again, affinity chromatography was performed using a 5 ml Streptrap column to remove cleaved protein from uncleaved protein, followed by a GSTrap column (GE Healthcare) to remove the 3C PreScission protease. The Vip3Aa protein was further purified by gel filtration chromatography with a ProSEC 16/60 6-600 HR SEC column (Generon, UK). SDS-PAGE was used to assess the purity of the fractions and the ones with the highest purity (>95%) were pooled and concentrated with a Vivaspin 20–30 kDa cut off filter (Sartorius, Germany) till 6 mg ml^−1^. Then the protein (protoxin) was aliquoted, frozen with N_2_(l) and stored. To prepare the activated toxin sample, 250 µl protoxin (at 6 mg ml^−1^) was incubated with 2.5 µl trypsin (at 6 mg ml^−1^), prepared in buffer 100 mM Tris-HCl pH 8.0, 500 mM NaCl, 5 mM MgCl_2_ and 2 mM DTT, for 2 h at 37 °C. Then the sample was frozen and stored as described above.

### Sample preparation for electron microscopy

For cryo-EM studies Vip3Aa was diluted (to a final concentration of 67.3 µg ml^−1^) in 0.1 M Tris (pH 8.0), 0.5 M NaCl, 5 mM MgCl2, 2 mM DTT and 0.01% NP40. After dilution, 3 µl of sample were applied to Quantifoil R2/2 300 mesh grids coated with a second layer of homemade thin continuous carbon, previously glow discharged in a GlowQube (25 mA, 10 s). After 1 min incubation, grids were blotted for 2 s and blot force of −20 using FEI Vitrobot Mark IV at room temperature and plunged into liquid ethane. Flash-frozen grids were subsequently stored in liquid nitrogen. Trypsin digested Vip3Aa was prepared similarly, using a slightly different protein concentration (75.2 µg ml^−1^) and grid type (quantifoil R2/1 300 mesh).

### Electron microscopy data acquisition

Cryo-EM grids were pre-screened in a JEOL 1230 and in a FEI Talos Artica microscopes equipped with a TemCam-F416 (TVIPS, Gauting, Germany) and a Falcon III (FEI) camera, respectively. High-resolution data were collected on a Titan Krios electron microscope operated at 300 kV, and images were acquired using a Gatan K2 Summit direct electron detector, operated in electron counting mode. For undigested Vip3Aa, EPU (Thermo) was used to record 3511 micrographs at a defocus range of −1.0 to −3.0 μm, with a magnification of 1.048 Å px^−1^ at the specimen level, and a total dose of 56.6 e- A^−2^ accumulated over 12 s and fractionated across 40 frames. For the digested Vip3Aa, 3090 micrographs were collected at a defocus range of −1.0 to −2.9 μm, with a magnification of 1.055 Å px^−1^ at the specimen level, and a total dose of 60 e- A^−2^ accumulated over 11 s and fractionated across 44 frames.

### Image processing

For both datasets, frames were aligned using MOTIONCOR2^52^, which also generated drift-corrected summed images with and without electron-dose weighting. Micrographs were manually inspected to remove pictures that contained crystalline ice or other forms of visible contamination, resulting in a final dataset of 3411 micrographs for the undigested and 3090 for the digested sample. CTF parameters were estimated with GCTF^53^ using the non dose-weighted images. Micrographs were picked with GAUTOMATCH, binned by two, and subjected to 2D classification using RELION 3^54^. The resulting 2D averages were then used as templates to pick again the entire dataset with GAUTOMATCH to generate initial particle stacks (729,405 Vip3Aa and 697,393 digested Vip3Aa particles). After one round of 2D classification, only averages showing high-resolution features were retained, which resulted in 478,797 for the undigested sample (protoxin from now on). 2D classification of the trypsin treated sample revealed a mixture of two conformations; some molecules (93,875 particles) remained in a configuration similar to the protoxin, whereas the majority (193,153 particles) adopted a distinct extended structure (toxin from now on) reminiscent to some elongated negative stained particles reported in earlier studies^13^.

Initial models were calculated using the SGD algorithm^55^ implemented in RELION 3. These reconstructions were low-pass filtered to 40 Å and used as starting model for 3D refinement with RELION 3 using different symmetries (C1, C2, and C4), revealing that the protoxin contained symmetry C2 whereas the toxin showed clear C4 symmetry. The unbinned particles were then re-centered and re-extracted, and subjected to a round of 3D refinement CTF and beam tilt refinement and particle polishing as implemented in RELION 3. After performing the Bayesian polishing, the particles were analyzed using 3D classification using six classes and without imposing symmetry. The protoxin did not show mayor signs of heterogeneity and all the particles (478,797) were included into a final 3D refinement round, using C2 symmetry and a spherical mask, that resulted in a 2.9 Å reconstruction by gold-standard FSC at 0.143^56^ (resolution and B-factor were calculated using a mask generated from a low-pass filtered map that followed the contour of the protein). In the case of the toxin, only the particles assigned to the class showing high-resolution features were selected (92,303 particles), and subjected to a 3D refinement run, imposing symmetry C4, that yielded a 2.9 Å reconstruction.

The quality of the density for most parts of the reconstructions was excellent. However, the four appendices that extend from the core of the structure, and that resulted to be the C-terminal domains (III–V), appeared to be not as well resolved, which hampered the subsequent model building efforts of the last two domains. We, therefore, decided to perform a round of symmetry expansion, density subtraction (to erase from the particles the density corresponding to domains I and II), and focused 3D classification without alignments (*K* = 6, *T* = 400). The result from the classification revealed that the last three domains move indeed as a rigid-body. The density showing better quality and best connectivity was used to build the atomic model of domains IV and V. This procedure gave nearly identical results for the protoxin and toxin datasets.

### Model building and refinement

The atomic models of domain I, II, and III were built de novo into the globally sharpened EM maps, using COOT^57^, for both the toxin and protoxin structures. The models were then subjected to iterative rounds of model building and real-space refinement with COOT and PHENIX using NCS constrains, and Ramachandran, rotamer, geometry, and secondary structure restraints^57,58^. To build domains IV and V, the Robetta server^59^ was used to generate an initial model (the recently determined structure of Vip3B, PDB 61V1, was automatically selected as a reference). The homology model of these two C-terminal domains was then docked into the best model obtained during the focused classification and subjected to several rounds of manual model building and real-space refinement. Next, domains IV and V were placed in all the subunits of the tetramers using the symmetry operators to generate the full-length structures. Finally, the complete tetramers were subjected to an ADP-only refinement cycle into the overall reconstructions (global sharpening values of −64 and −69 Å^2^) to allow the temperature factors of the domains IV and V to change slightly to better reflect the flexibility of this part of the molecule. In the case of the protoxin, the structure was modeled from residue 14 to 789, with the exception of the solvent-exposed trypsin cleavage loop (residues 190–202). For the toxin, the atomic model was built from residues 95 to 789, although 2D and 3D analysis showed that the coiled coil region extends until residue ~45 and only the most N-terminal amino acids appear to be disordered. The internal diameter of the coiled-coil region of toxin was estimated with HOLE^60^, and figures where generated with CHIMERA^61^ and CHIMERAX^62^.

### Reporting summary

Further information on research design is available in the Nature Research Reporting Summary linked to this article.

## Supplementary information

Supplementary Information

Peer Review File

Supplementary Video 1

Supplementary Video 2

Supplementary Video 3

Supplementary Video 4

Reporting Summary

Source Data

## Data Availability

Data supporting the findings of this manuscript are available from the corresponding authors upon reasonable request. A reporting summary for this article is available as a Supplementary information file. Source data are provided with this paper. The cryo-EM densities (including sharpened and unsharpened maps, masks, focused classification reconstructions and half-maps), and atomic coordinates for the protoxin and toxin Vip3Aa structures have been deposited in the EMDB: EMD-10492 and EMD-10493, and PDB 6TFJ [10.2210/pdb6TFJ/pdb] and PDB 6TFK [10.2210/pdb6TFK/pdb], respectively.
