# Supplementary material for: Molecular architecture and activation of the insecticidal protein Vip3Aa from Bacillus thuringiensis
Source: Nat Commun. 2020 Aug 7;11:3974. doi: 10.1038/s41467-020-17758-5 (PMC7414852; doi:10.1038/s41467-020-17758-5)
Supplement: Supplementary file 1 — Supplementary Information [file 41467_2020_17758_MOESM1_ESM.pdf]

Supplementary Information

Molecular architecture and activation of the insecticidal protein Vip3Aa from *Bacillus thuringiensis*.

Núñez-Ramírez et al.

Supplementary Tables

Supplementary Table 1. Cryo-EM data collection, refinement and validation statistics

	Protoxin (EMD-10492) (PDB 6TFJ)	Toxin (EMD-10493) (PDB 6TFK)
**Data collection and processing**		
Microscope	Titan Krios (DLS)	Titan Krios (DLS)
Voltage (kV)	300	300
Electron dose (e/A^2)	56.6	60
Dose rate (e/A^2/fraction)	1.41	1.36
Detector	K2 Summit	K2 Summit
Defocus range (µm)	0.5-3.0	0.6-2.9
Calibrated pixel size (Å)	1.048	1.055
Micrographs	3511	3090
Total extracted particles (no.)	729,405	697,393
Final particle images (no.)	478,797	92,303
Symmetry imposed	C2	C4
Resolution (per 0.143 FSC) (Å)	2.9	2.9
Applied B-factor (Å^2)	-69	-64
**Refinement**		
RMS deviations		
Bond lengths (Å)	0.002	0.002
Bond angles (°)	0.423	0.466
Ramachandran (%)		
Outliers	0.00	0.00
Allowed	8.38	9.12
Favored	91.62	90.88
Rotamer outliers (%)	0.11	0.24
MolProbity score	1.87	1.92
Clashscore	6.70	7.17

Supplementary Figures

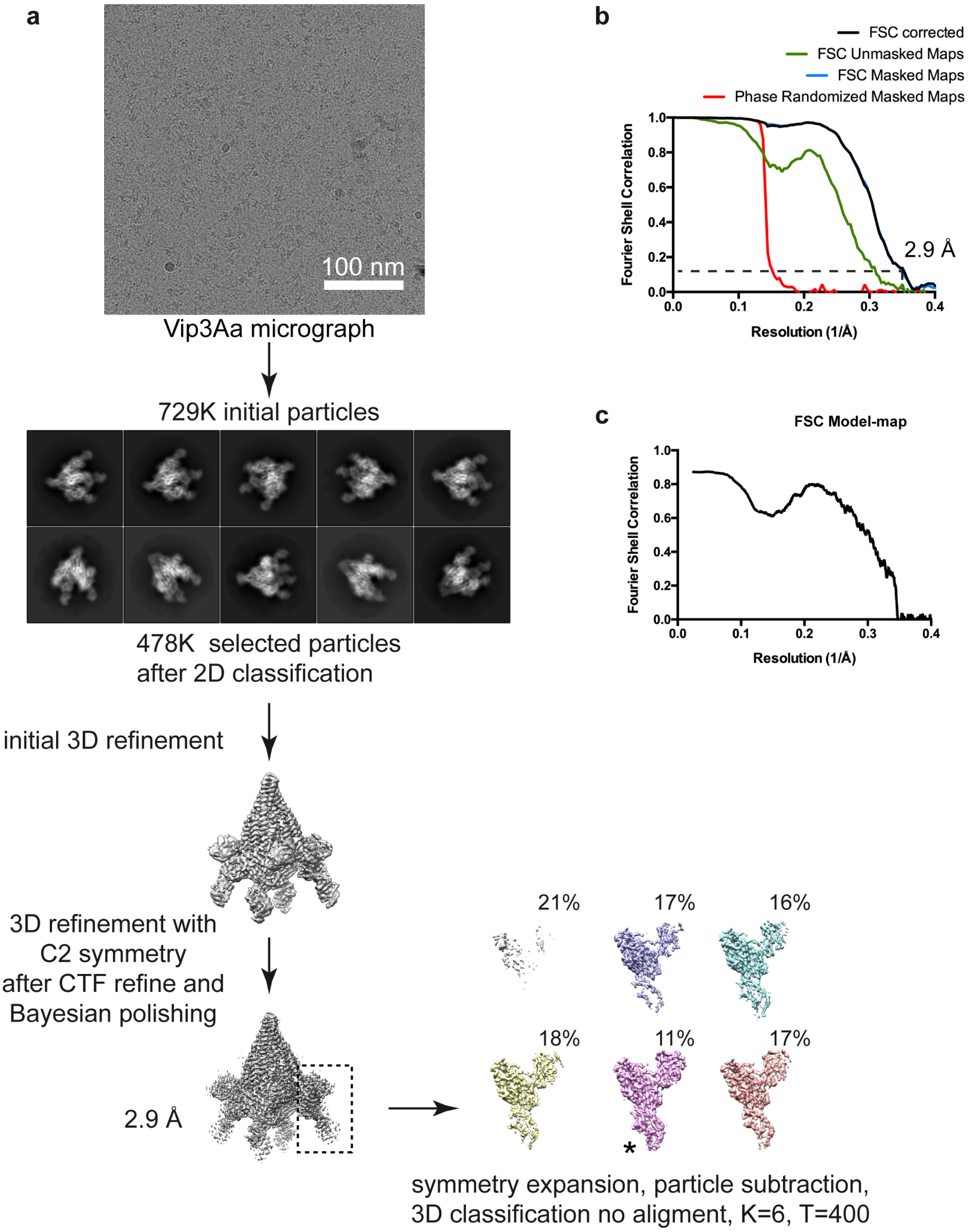

Supplementary Figure 1. Cryo-EM image processing of *Bacillus thuringiensis* Vip3Aa protoxin. **a,** Representative micrograph, from a dataset containing 3511 images, and workflow of image processing for the protoxin particles. A focused classification approach was used to resolve the flexibility present in the C-terminal region of the protein. Inner and outer monomers of the tetramer were classified

independently, giving nearly identical results (only one is shown for clarity). Reconstruction used for model building of domains IV and V is marked with an asterisk. **b,** Fourier Shell Correlation of the final density map. Source data are provided as a Source Data file. **c,** Map to model Fourier Shell Correlation. Source data are provided as a Source Data file.

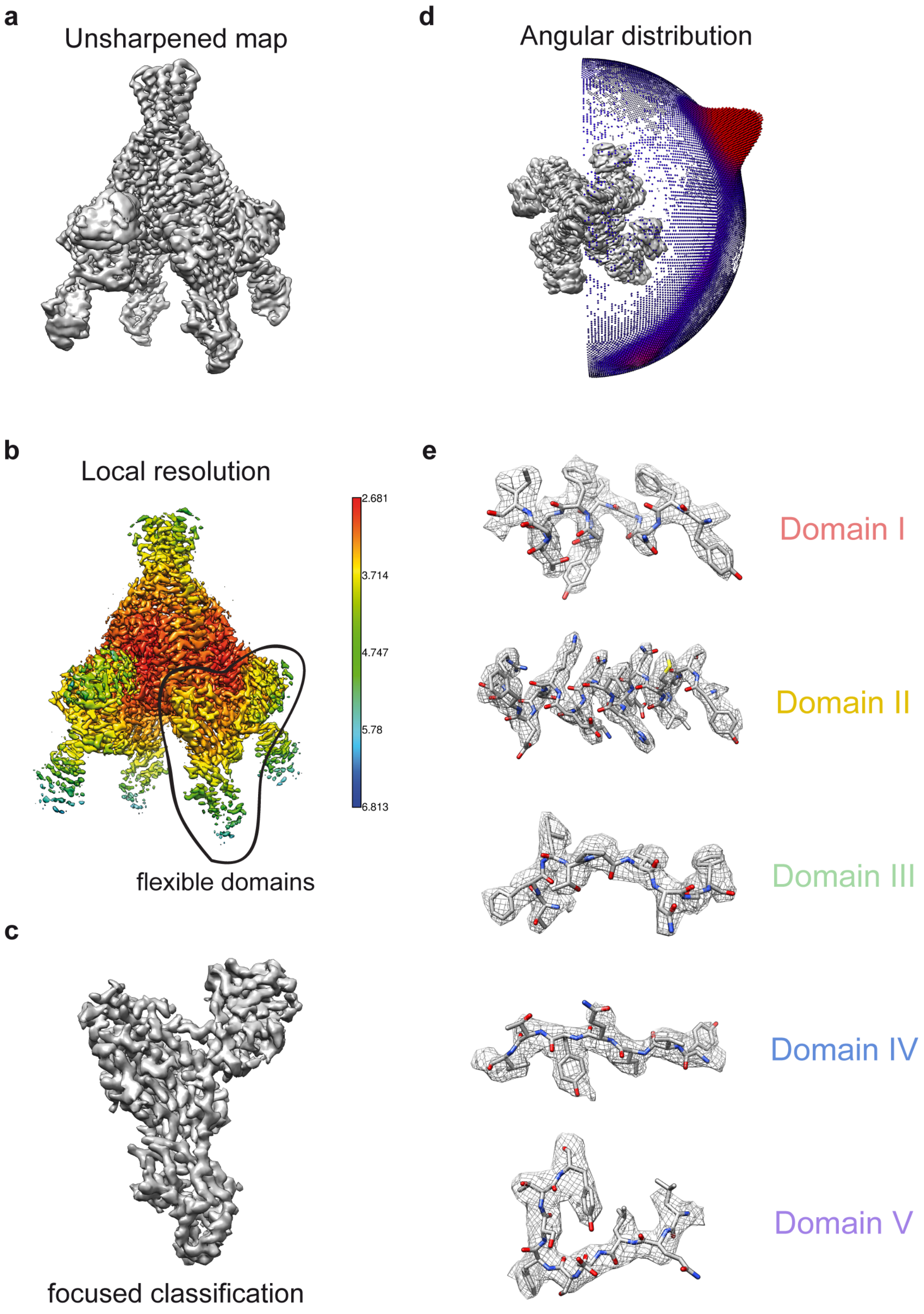

Supplementary Figure 2. Analysis of the Vip3Aa cryo-EM reconstruction. a, Unsharpened map of the Vip3Aa protoxin showing clear density for all the domains of the protein. **b,** Local resolution of the globally sharpened map of the Vip3Aa protoxin. The region corresponding to domains IV and V appears particularly flexible.

c, Focused classification map used for the building of the atomic structure of domains IV and V. **d,** Angular distribution plot showing the range of orientations observed for the protoxin. **e,** Close-up views from all Vip3Aa domains showing the model to map fit.

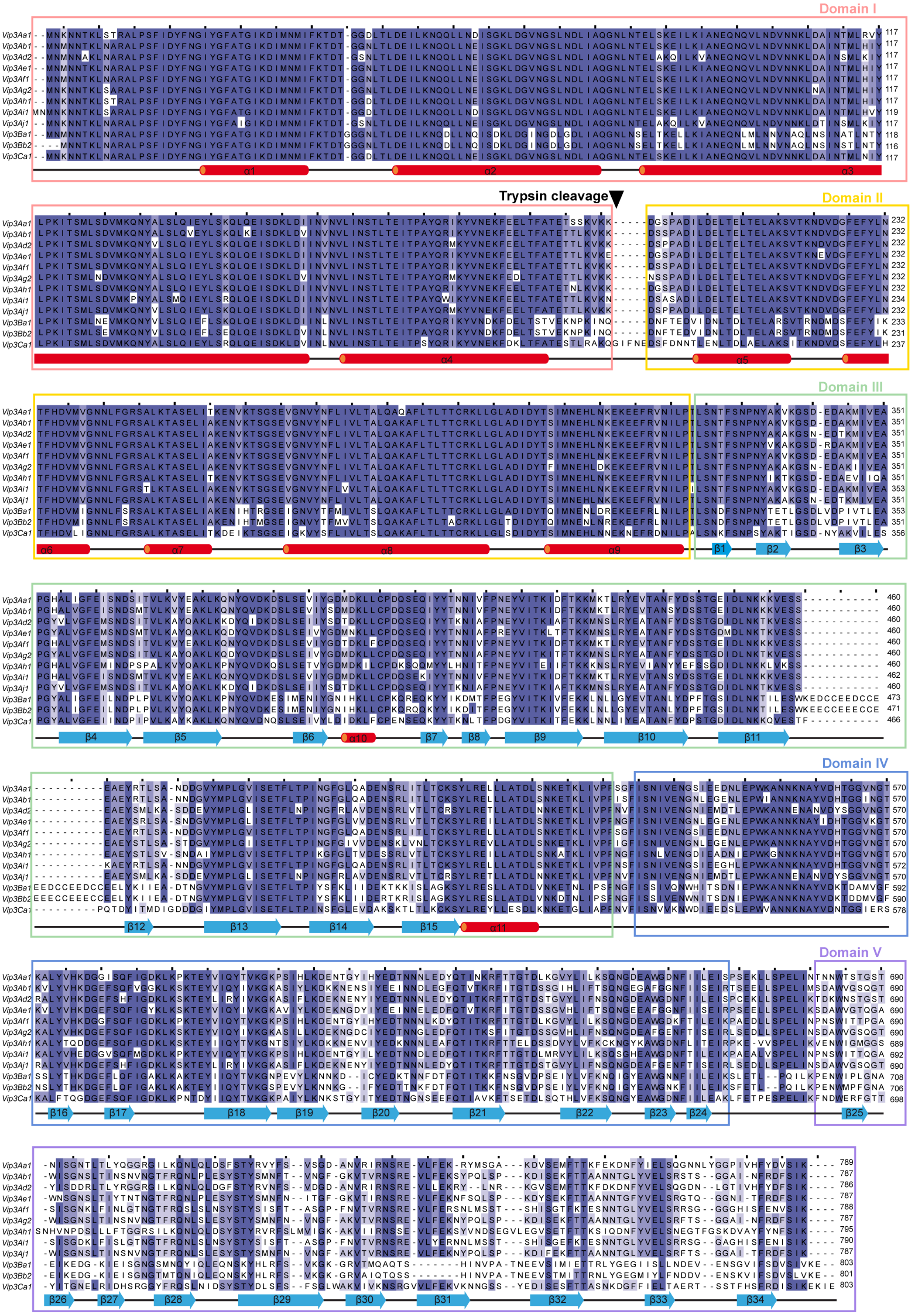

Supplementary Figure 3. Multiple sequence alignment of Vip3 family members.

The protein sequences used are as follows: Vip3Aa1 (GenBank accession number AAC37036), Vip3Ab1 (AAR40284), Vip3Ad2 (CAI43276), Vip3Ae1 (CAI43277),

Vip3Af1 (CAI43275), Vip3Ag2 (ACL97352), Vip3Ah1 (ABH10614), Vip3Ai1 (KC156693), Vip3Aj1 (KF826717), Vip3Ba1 (AAV70653), Vip3Bb2 (ABO30520), and Vip3Ca1 (ADZ46178). Domains are highlighted in boxes colored as in **Fig. 1c**. Secondary structure assignments refer to the Vip3Aa protoxin structure determined here.

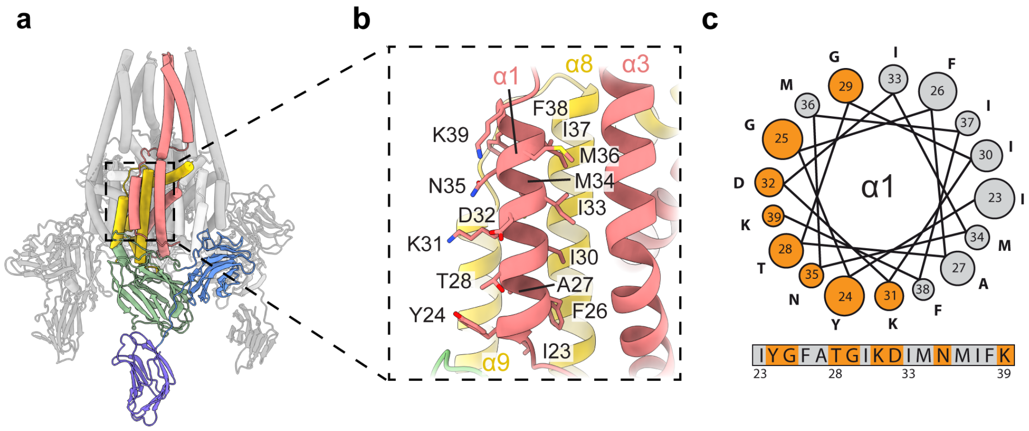

Supplementary Figure 4. Vip3Aa α1 shows a highly amphipathic amino acid distribution. **a,** Side view of the Vip3Aa tetramer. One monomer has been colored according to the domain organization (domains colored as in **Fig. 1c**). **b,** The first alpha helix is highly amphipathic. The hydrophobic region docks against the core of the tetramer in a cavity formed by helices α3, α8 and α9. **c,** Scheme of the α1 amino acid distribution colored based on their hydrophilic nature (orange, polar; grey, non-polar).

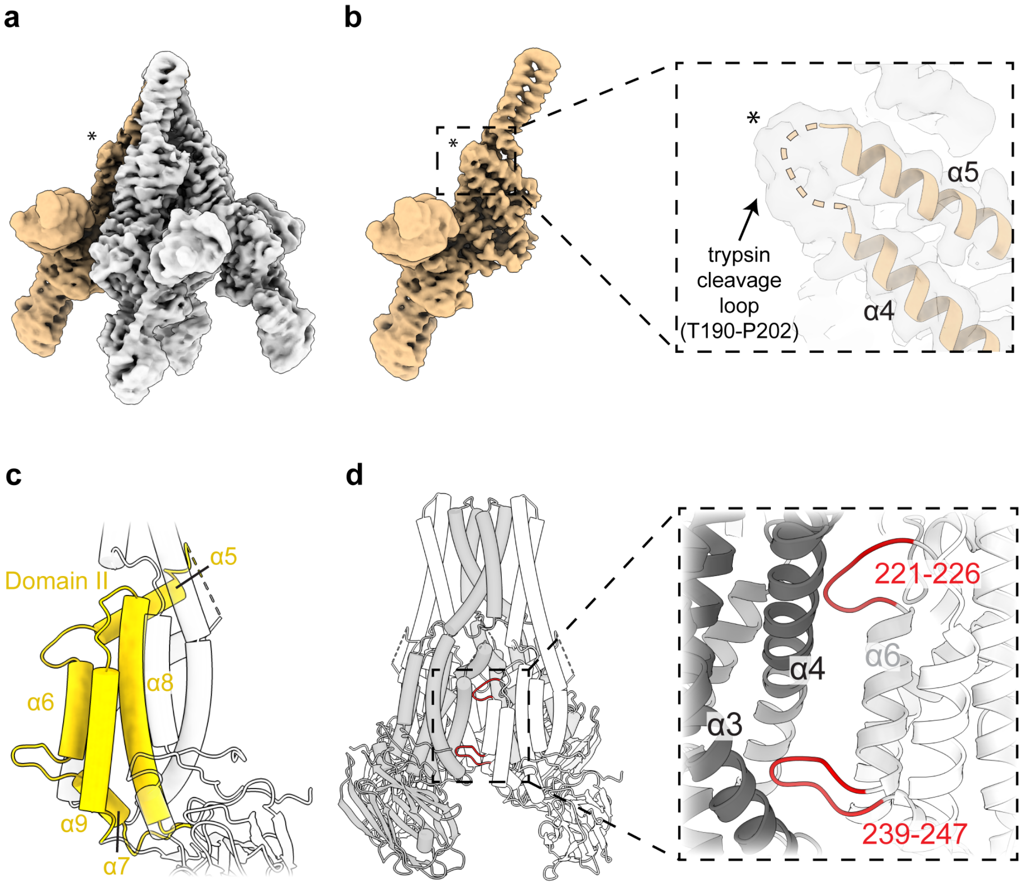

Supplementary Figure 5. The central domain II helps to stabilize the Vip3Aa oligomer. **a,** The trypsin cleavage sites extend out from the core of the protoxin and are fully accessible (one loop is marked with an asterisk). **b,** Detailed view of the protease cleavage loop. Although the flexibility prevented modeling at atomic level, there is clear density for the entire loop in the unsharpened EM map. **c,** Domains II is composed of five alpha helices. **d,** Two extended loops (shown in red) project from domain II towards the neighboring monomer to stabilize the Vip3Aa tetramer. Domains IV and V have been removed for clarity.

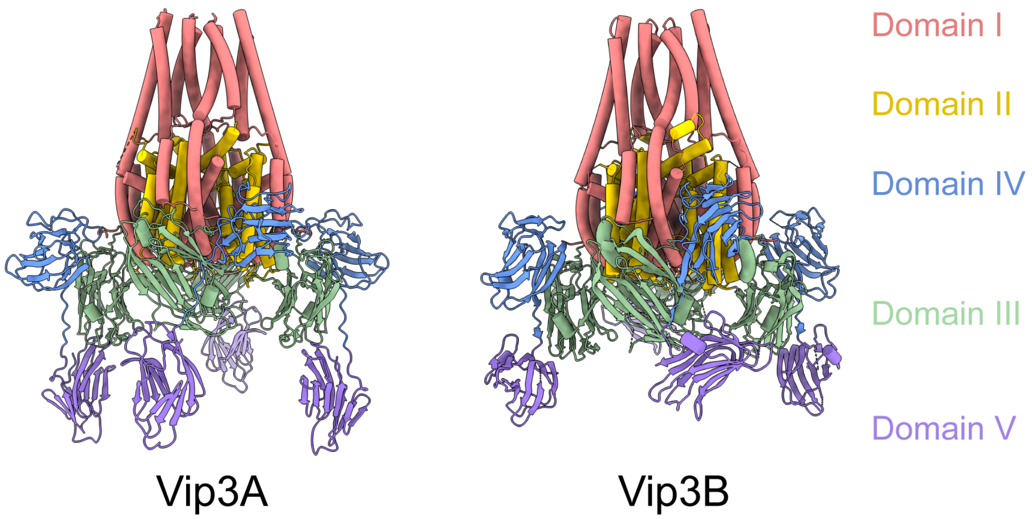

Supplementary Figure 6. Structural comparison of the Vip3A and Vip3B protoxins. Similar views of the Vip3A protoxin determined in this work and the crystal structure of the Vip3B protoxin (PDB ID 6V1V) colored by domain organization.

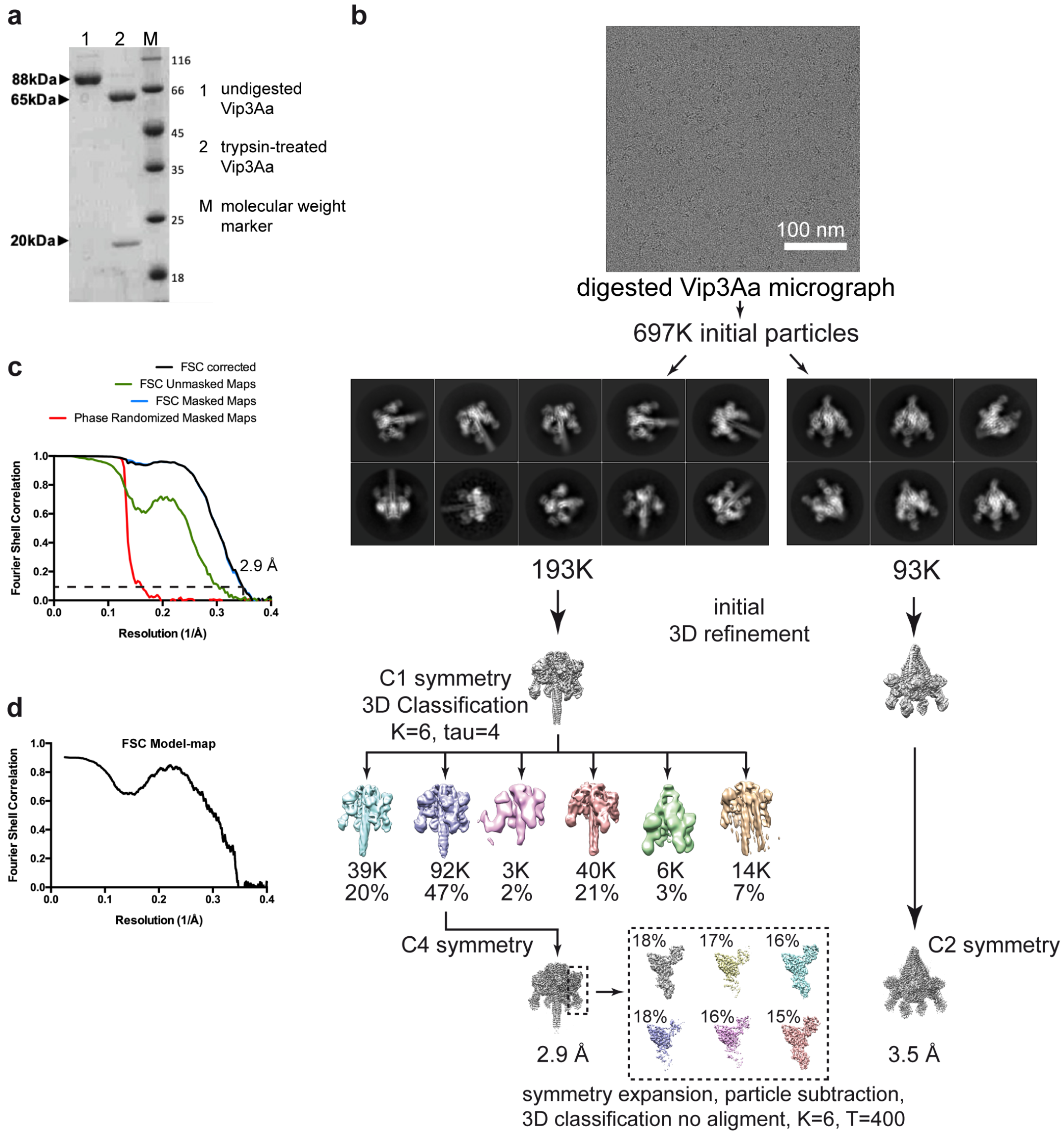

Supplementary Figure 7. Cryo-EM image processing of the trypsin digested Vip3Aa sample. **a,** SDS-PAGE gel of the undigested and trypsin-treated Vip3Aa samples. This experiment was repeated independently at least three times. Source data are provided as a Source Data file. **b,** Representative micrograph, from a dataset containing 3090 images, and workflow of image processing for the trypsin treated sample. Approximately one third of the particles were grouped in 2D classes that share a remarkable similarity to those seen in the undigested protein. These particles produced a 3.5 Å reconstruction that was virtually indistinguishable from that of the

protoxin at this resolution. The rest of the particles were further processed and resulted in a 2.9 Å reconstruction of the toxin. Focused classification was performed in the C-terminal region of the protein to improve the heterogeneity found in this area. **c,** Fourier Shell Correlation of the 3D reconstruction of the toxin. Source data are provided as a Source Data file. **d,** Map to model Fourier Shell Correlation. Source data are provided as a Source Data file.

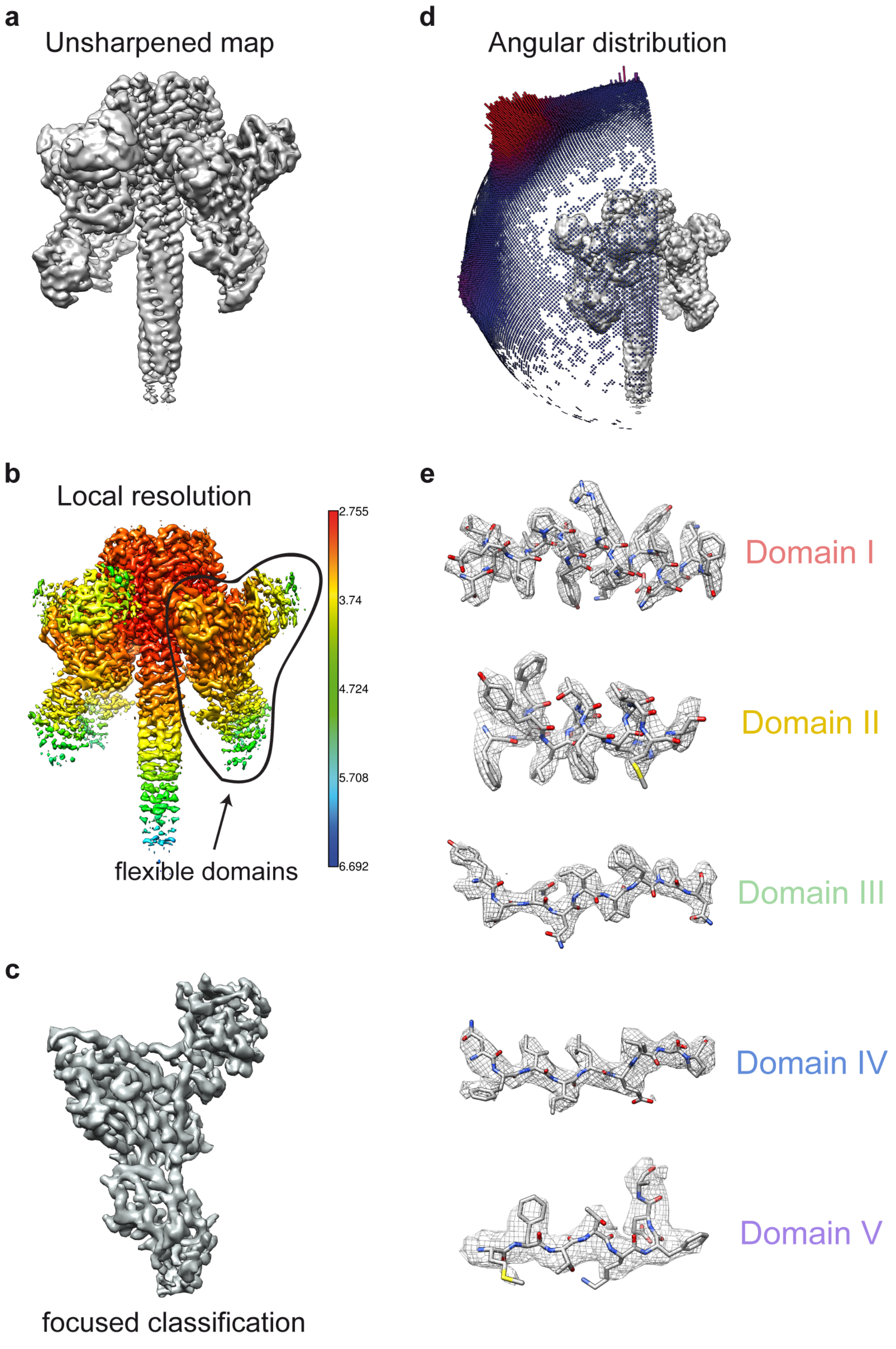

Supplementary Figure 8. Analysis of the Vip3Aa cryo-EM reconstruction. a, Unsharpened map of the Vip3Aa toxin showing density for all protein domains. **b,** Local resolution of the final, globally sharpened map. Similarly to the protoxin, the

region corresponding to domains IV and V appears particularly flexible. **c,** One of the best resolved focused classification maps that shows that the structure and orientation of the C-terminal domains remains unchanged after trypsin treatment. **d,** Angular distribution plot showing the range of orientations observed for the toxin. **e,** Close-up views from all Vip3Aa domains showing the model to map fit.

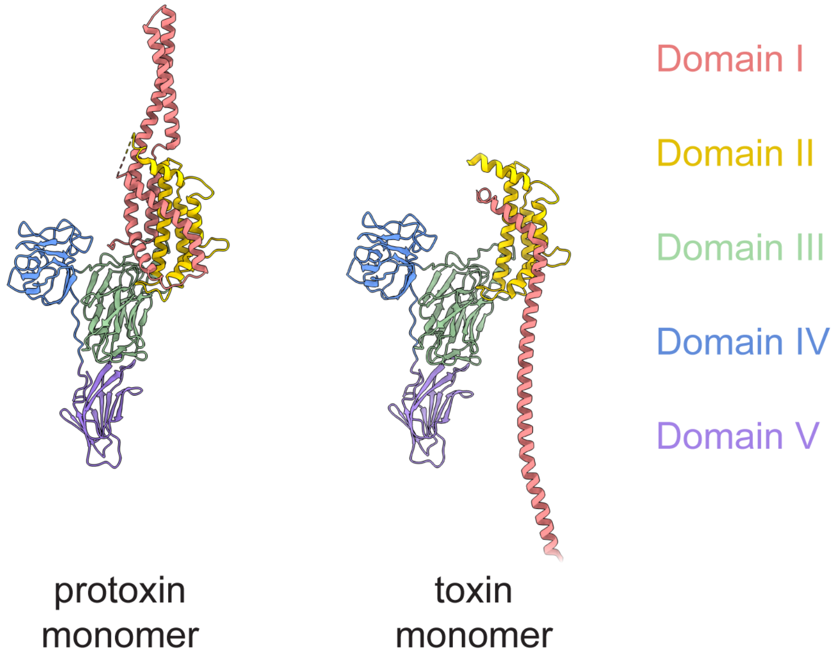

Supplementary Figure 9. Comparison of Vip3Aa domains prior and after trypsin digestion. Individual protoxin and toxin monomers colored by domain organization. Domains II-V maintain the same configuration in both states.
