# Supplementary material for: Molecular architecture and activation of the insecticidal protein Vip3Aa from Bacillus thuringiensis
Source: Nat Commun. 2020 Aug 7;11:3974. doi: 10.1038/s41467-020-17758-5 (PMC7414852; doi:10.1038/s41467-020-17758-5)
Supplement: Supplementary file 2 — Peer Review File [file 41467_2020_17758_MOESM2_ESM.docx]

Peer Review Information

**Journal:** Nature Communications

**Manuscript title:** Molecular architecture and activation of the insecticidal protein Vip3Aa from *Bacillus thuringiensis*.

**Corresponding author name(s):** Dr Ernesto Arias-Palomo

**Editorial notes:**

**none**

**Reviewer comments & decisions:**

**Reviewer comments, first version:**

Reviewer #1 (Remarks to the Author):

This is a lovely structural biology paper describing the prepore structures and pore structures of Vip3, an insecticidal pore forming toxin currently in use in biotech industry. The main point of this research is the postulation of a spring-loaded "folded cane" mechanism where by helices 1-4 of domain one stretch into one very long alpha helix at the opposite end of the protein complex. This mechanism was not predicted by the competing crystal structure of the prepore (Zheng et al, Protein Science 2019). Overall, this is a protein of high commercial importance to the agriculture industry. As the authors state, these two structures serve as a strong foundation for development of the protein. Top of the list of development would be to understand binding of the protein to the receptor as this is a common method of insect resistance to the insecticide. Understanding the mechanism of insertion also has significant value to the class of pore forming proteins.

My first criticism of the paper is that the structures of Domain IV and V are not resolved and these are important for target binding. This is potentially the Achille's heel of the protein with respect to insect receptor targeting/binding and is probably the point of insect resistance. Resolution of the Domain IV region and the gap relative to the neighbouring domain III is also critical. The "folding cane" hypothesis of pore formation looks logical but there needs to be some support that the alpha-helices can pass through this gap. Based on the figures, it looks like domain IV would sterically hinder this conformational change although based on the recently published structure then this might be OK.

Firstly, any additional effort in resolving the structures of these domains from the EM data should be made (see Pang et al., Nat comms, 2019 for potential ideas). However an alternative approach is to use the newly published crystal structure (PDB ID: 6V1V). It would still be preferential to use the EM data as there will be conformational information in how the domains move that would be of great value to this paper.

My second criticism is that there is a mechanism of action postulated based on the structures but this MOA is not tested via mutants. Although having said this, they are two very important structures that help explain much of the literature including published mutants which are cited. This is possible as the protein is expressed using E. coli expression system and there are cell based assays in at least Sf9. With the right mutation, this would be a good tool for demonstrating the "folding cane" hypothesis.

Page 5 paragraph 1; the sentence "Overall, the high-resolution structural features and the quality of the EM map have permitted unambiguous de novo atomic modelling of the protoxin and detailed structure and function analyses." is a little inaccurate. The authors should say that "Overall, the high-resolution structural features and the quality of the EM map have permitted unambiguous de novo atomic modelling of domains 1 - 3 of the protoxin" since not all of the pro-toxin was built using the map. And remove the comment about function analysis as only the structure was analysed (see comment about about testing the model using assays). Incorporation of some of the detailed structural analysis from Zheng paper would provide a more complete story.

Page 6 paragragh 2 line 6 "Domain III comprises residues 328-532 and contains three antiparallel β-sheets that form a β-prism fold strikingly similar to that found in members of the Cry insecticidal δ-endotoxins, and that has been shown to recognize the sugar moieties that decorate the receptors of the target cell".
There are know beta-prism domains associated with other pore forming protein domains that are not demonstrated to bind to glycans (GNIP, Plu-MACPF). Take care not to imply glycan binding due to the existence of a beta-prism domain especially as there are no wet-lab experiments used to support this. I understand that it is implied not stated in the manuscript. Same comment for page 7 regarding domains IV and V. It is simply not known what they bind. In addition the crystal structure suggests a role or domain IV and V in ligand binding. And why is there the need for multiple binding sites?

Do the structures provide insight into if there is a level of control of the timing of protease activation? i.e. How is it prevented from pore formation prior to docking onto the receptor? How is pH regulation affected based on the structure?

There is comparison to Haemagglutinin (6 long helices?) and M2 class I (4 short helices) discussed. But how does the pore structure compare to the snap25/snare complex (4 long helices)?

Minor:
Abstract: replace C-term with C-terminal
Supp fig 1b and supp fig 6b: higher resolution and larger images of the 2d class averages would be much appreciated

Labelling the domains in fig 3c and b would be helpful to the reader.


Reviewer #2 (Remarks to the Author):

This manuscript describes the architecture of protoxin and toxin forms of Vip3Aa obtained using Cryo-EM studies. The resolution obtained is sufficient to confirm previous structural predictions and when combined with modelling approaches gives near atomic resolution. By far the most interesting aspect of the described work is the large structural difference observed between the native and activated forms.

As the authors will be aware while this manuscript was being considered by Nature Communications an alternative Vip3 protoxin structure was published in Protein Science which was derived from a combination of X-ray crystallography and molecular replacement techniques. The two structures appear very similar but clearly the authors need to compare the two.

What is needed to make this a standout paper (not just another protein structure paper) and therefore suitable for the broad readership of Nature Communications is some in depth insight as to how the observed structural rearrangement associated with activation relates to the biological properties of the protein. Although the authors have attempted to do this, I find many of the explanations rather vague and needing more detail. Particular areas that need further consideration are:

1) It is well established that these Vip3A toxins require activation for activity and this manuscript provides an explanation for that. The authors mention that multiple N-terminal cleavages take place including the removal of 11 amino acids at the very N-terminus as well as the cleavage at 198. The assumption is made that the 198 cleavage is the crucial one, which although consistent with the molecular shifts observed (eg Fig 4c), is not actually demonstrated. The authors should make specific reference to papers such as Zhang et al (https://doi.org/10.1016/j.ijbiomac.2017.09.101) who demonstrated that blocking this cleavage prevented activity. Ideally the authors should replicate this mutation and confirm that no structural rearrangement takes place.

2) Palma et al’s paper on Vip3Ag is referenced (ref 22) but the authors should comment on the fact that using size exclusion chromatography Palma et al concluded that there were no significant structural differences between protoxin and toxin. This could just be a limitation of SEC.

3) On page 7 it is mentioned that 30% of the activated molecules maintained a protoxin configuration. An obvious explanation would be that 30% of the proteins were not activated. Although the authors claim to have used conditions in which full activation was achieved I could not find any data (eg SDS gel) to confirm this. If full activation was achieved then I would like to see some discussion as to why such a high percentage of molecules did not “flip”. This also relates to point 4 below.

4) What the readership of this manuscript will really want to know is how this structural rearrangement reflects the biological activity of the protein. Although on page 3 is it noted that many other mechanisms other that pore-formation have been proposed, the rest of the manuscript seems to assume a pore-formation model. If I have misjudged this then I recommend that the authors also need to consider how the structure might relate to such other effects - including acting internally (https://doi.org/10.1371/journal.ppat.1007347 ). Assuming a pore-formation model though, then the model presented in Fig 5 requires further explanation. In this model structural rearrangement occurs following receptor binding yet in this manuscript it occurs (at least for 70% of the molecules) following protease treatment alone. The manuscript states that this process could be further stimulated by other factors like the interaction of with membrane receptors. This is not convincing – evidence indicates that receptor binding is not required for activation – not only in the in vitro studies in this manuscript but also in previous work where activated toxins could form pores in planar lipid bilayers. The authors need either to provide additional experiments to confirm that receptor binding does in fact further stimulate the activation process or they need to refine their Fig5 model. There are still many people working with Bt toxins who believe that receptors do not play a crucial role in activating the proteins – but simply act to concentrate them on the cell surface in the correct orientation and in sufficient numbers to have an effect.

5) Assuming once more a pore-formation model I feel that more clarity is required on what the nature of that pore will be. It is not clear to me whether the authors favour a model in which the helical bundle itself would allow ion movement which could then be followed by water via other means (eg aquaporins) to produce swelling/lysis, or whether the bundles would oligomerize to form a pore, or just whether the amphipathic helix 1 would oligomerize to form a pore. More discussion on these options is required

Finally there are some minor points which should be considered
i) On page 7 the structure prediction data can now be enhanced by the Zheng et al publication in Protein Science with respect to the carbohydrate binding domains
ii) Page 9 are N@d layers real or a typo?
iii) The last line of the manuscript is commonly thrown about – the authors should give some indication of how they believe that their data could be used to engineer proteins with increased activity. Which bits would be engineered and why?
iv) Many of the references require italicization.


Reviewer #3 (Remarks to the Author):

Núñez-Ramírez et al. present the first structures of prepore (latent, protoxin) and pore (activated, toxin) states of the Vip3Aa system of the VIP3 family of proteins - an important system in agricultural science as a genetically encoded pesticide against Lepidopteran pests. Their study draws upon comparisons between two cryo-electron microscopy single particle reconstructions of the latent pro-toxin state with a trypsin-activated toxin (pore) form. Núñez-Ramírez et al. further provide a detailed analysis of the structural changes that occur between these forms and propose a model to describe the mechanism of these structural changes. This study provides a robust basis upon which to understand previously described mutations and effects of the Vip3Aa, thereby aiding the development of improved pesticidal proteins, as well as helping to shed light upon the mechanism/protein fold employed by this particular family. Overall the manuscript is well written and easy to understand, with a logical and fair presentation of the data.


Major comments:

1. The authors describe two extended loops in domain II (residues 221-226, 239-247) that supposedly stabilise the oligomer, how was this determined? Were any efforts made to assess the effects of these loops on the stability i.e. by truncating these to glycine or is this inferred solely from the structure? These loops are not clear in Fig. 1d.

2. Figure 4b: “there is a clear peak of density coordinated by four symmetry related asparagine” - while the atomic model illustrates this, it would be helpful to guide readers if there were a map/model overlay that shows the quality of fit.

3. In fact, all maps shown in main figures alongside models appear to be the unsharpened maps. It is difficult to assess the agreement between the models and the EM density at the level of main chain. I would suggest a supplementary figure that shows map-to-model fit for different domains of the structure. A map-to-model Fourier Shell Correlation would also be advisable to demonstrate that over fitting has not occurred - this could be plotted on the same axis as Supp Fig 1c and 6c.

4. Methods: The authors state that they use a spherical mask for the final refinement, but I don't think they mean this, or at least it isn't clear to me. Usually one would use a mask generated from a low pass filtered map (this is why in sup fig 6 the red curve is present, to assess whether this mask introduces correlated information into the half sets i.e. interfering with the refinement). Surely, a low pass filtered protein mask was used, rather than a sphere (as one excludes more solvent). If a spherical mask was used for refinement, that's fine, but the resolution would underestimated and sharpening would likely create a lot of noise.

5. In two of the figures (sup fig 1f and sup fig 6f) the authors show maps that have regions that appear over sharpened (domains IV and V), generating noise. The core region is perfectly fine to model, but evidently domains IV and V at that sharpening value are not possible to model. Of course the authors acknowledge this - so that’s fine. Did the authors attempt to fit their homology models of domain IV and V generated from Robetta into any of the maps (sharpened, unsharpened, filtered, etc.)? Were different sharpening values used for modelling or just one? Were the homology models refined against any maps (the unsharpened map, I note, still possesses distinct features that would enable fitting e.g. the connecting loop)? I suspect with a different sharpening value one could probably fit the homology models into the map presented.

6. Can the authors clarify in their methods for model building:
- What sharpening values were applied for model building? Did these differ from those reported in table 1?
and
- Whether they built into one or more globally sharpened map(s), or,
- Whether they built into a locally filtered and globally sharpened map, or,
- Whether they built into a locally sharpened and locally filtered map.

7. If the authors only built into one map at one sharpening value, I would recommend reducing the sharpening that was used when considering domains IV and V, as this may allow further details and features to be observed. Alternatively some useful tools would be local_deblur in Scipion, relion_locres which produces a locally filtered map, or a locally sharpened map from phenix.autosharpen - these are useful when there is a large variation in local resolution. This may enable more confident fitting of domains IV and V.

8. Lastly the authors have only deposit two EM maps. Personally, I feel the following maps should be uploaded. This benefits the community by enabling transparency and validation of the map, including assessment of model-to-map fit.
- The unsharpened map (1)
- The sharpened maps (1) used for building (whether this is globally sharpened, locally sharpened and/or locally/globally filtered - it doesn't matter, it's necessary to compare the models against). Multiple different sharpened maps or segmented maps may be uploaded e.g. focused refinements,
- The mask (1) used for the refinement/sharpening - to ensure the masks were not too tight.
- The half maps (2) enabling the FSC to be calculated i.e. to verify the resolution.

That would amount to five maps per structure. I would encourage the authors to consider depositing these.

Minor comments:
- Can the authors provide an SDS-PAGE to show the purity of the final recombinant Vip3a protein?
- Abstract uses abbreviations “N-term” and “C-term” - use full terminology.
- Abstract “biotec” should be “biotech”?
- Figure 1 caption typo/unclear: “Inset displays the same orientation the EM map with the domains IV and V shown in grey”
- Methods: “[...] elongated negative staining particles reported [...]” should be “negative stained”
- Discussion: Suggest rephasing double negative “Not only domain I is not eliminated by protease digestion [...]” to “Not only is domain I retained after protease digestion [...]”
- Discussion - suggest rephrase: “[...] maintain fundamentally same contacts in the protoxin and toxin [...]” to “[...] maintain fundamentally the same contacts in the protoxin and toxin [...]”
- Discussion - suggest rephrase: “[...] F229, which locates at α6 [...]” to “[...] F229, which is located at α6 [...]”
- Supplemental figure 7 could be accompanied by a separate model superpositions of domains I, II and III in the pro- and toxin forms to help illustrate the conformational changes (or lack of) in each region of the complex.
- Figure 4g: “g” bold font
- Sup Fig 1b caption: “Workflow of image processing the protoxin particles.” rephrase “Workflow of image processing for the protoxin particles.”
- Sup Fig 2 caption: “Domains a highlighted in boxes colored as in Fig. 1a.” should be “Domains are highlighted in boxes colored as in Fig. 1a.”
- Figure 1 and 3 should have scale bars (I note the scale bar on the 2D class average), however it is difficult to assess the dimensions of the complex.
- Figure 2 caption typo/unclear:
a) Domain should not be plural: “domains III forms [...]” should be “domain III forms [...]”
b) “Groove” is misspelt.

**Author rebuttal, first version:**

Reviewer #1 (Remarks to the Author):

This is a lovely structural biology paper describing the prepore structures and pore structures of Vip3, an insecticidal pore forming toxin currently in use in biotech industry. The main point of this research is the postulation of a spring-loaded "folded cane" mechanism where by helices 1-4 of domain one stretch into one very long alpha helix at the opposite end of the protein complex. This mechanism was not predicted by the competing crystal structure of the prepore (Zheng et al, Protein Science 2019). Overall, this is a protein of high commercial importance to the agriculture industry. As the authors state, these two structures serve as a strong foundation for development of the protein. Top of the list of development would be to understand binding of the protein to the receptor as this is a common method of insect resistance to the insecticide. Understanding the mechanism of insertion also has significant value to the class of pore forming proteins.

We thank the referee for their positive view and kind comments.

My first criticism of the paper is that the structures of Domain IV and V are not resolved and these are important for target binding. This is potentially the Achille's heel of the protein with respect to insect receptor targeting/binding and is probably the point of insect resistance. Resolution of the Domain IV region and the gap relative to the neighbouring domain III is also critical. The "folding cane" hypothesis of pore formation looks logical but there needs to be some support that the alpha-helices can pass through this gap. Based on the figures, it looks like domain IV would sterically hinder this conformational change although based on the recently published structure then this might be OK.

Firstly, any additional effort in resolving the structures of these domains from the EM data should be made (see Pang et al., Nat comms, 2019 for potential ideas). However an alternative approach is to use the newly published crystal structure (PDB ID: 6V1V). It would still be preferential to use the EM data as there will be conformational information in how the domains move that would be of great value to this paper.

We share the reviewer's view on the relevance of domains IV and V in target recognition and insect resistance. We have attempted to produce several constructs containing either isolated or multiple C-terminal domains to perform crystallization studies. However, this approach did not provide positive results. Interestingly, although the initial efforts to build an atomic model based on the EM reconstructions were not successful, we have now performed particle subtraction and focused 3D classification, which has significantly improved the EM density of the C-terminal region and has allowed us to determine the atomic structure of these domains.

Thus, we now present the complete structures of Vip3A in the protoxin and toxin states. The structural analysis of domains IV and V reveals that they indeed belong to the CBM family and show similarity to glycoside hydrolases, according to the DALI server (Holm L Bioinformatics 35 2019). In light of this result, we have rewritten the paragraph describing these domains.

Moreover, although the orientation of the C-terminal domains remains basically unchanged between the protoxin and toxin configurations, detailed examination of the result from the 3D classification shows that these domains posses some degree of flexibility and that they can move as a rigid body (**new Fig. 2c and Supplementary Video 3**). Interestingly, comparison with the newly published structure of Vip3B, whose C-terminal domains share ∼40% sequence identity and overall folding, reveals a significant a change in the disposition of domain V (rotates 99ϒ and shifts 1.6 Å according to Dyndom, Lee RA et al. Bioinformatics, 19, 10, 2003) (**new Fig. 2d**). Taken together, these results confirm the flexible nature of these domains that is likely relevant for the function of the protein. We have now included in the revised manuscript and new figures the comparison between Vip3A and Vip3B that highlights the movement of these domains.

Regarding the folding cane hypothesis, we agree with the reviewer that the figures should illustrate that the α-helices can pass through the gap left between monomers and we apologize if they were unclear. The minimum distance of the gap located between domains III and IV of adjacent monomers is ~14 Å (see Figure below), which would be sufficient to accommodate the passage of a single helix. We now indicate the distance of this gap in the **new Fig. 1d**. Moreover, the flexibility of these domains can broaden the cleft, further facilitating the conformational change that leads to the active form.

**Domain IV**

**Domain V**

**Domain III**

**GAP**

**Figure. Representation of the gap between C-terminal domains of adjacent monomers.** The α-helix from domain I (in pink) can pass through the C-terminal domains during the activation process. Domain III, IV and V, represented as surface, define the boundaries of the gap between two adjacent monomers.

My second criticism is that there is a mechanism of action postulated based on the structures but this MOA is not tested via mutants. Although having said this, they are two very important structures that help explain much of the literature including published mutants which are cited. This is possible as the protein is expressed using E. coli expression system and there are cell based assays in at least Sf9. With the right mutation, this would be a good tool for demonstrating the "folding cane" hypothesis.

We agree with the reviewer on the interest of further investigating the molecular details of the proposed model. We have already produced several mutants and we plan to perform future experiments to interrogate the mode of action of this protein as soon as the lockdown measures that have been taken around the world to palliate the effects of covid-19 allow it. Importantly, however, this model is currently supported by previous mutagenesis data. Some of the mutants discussed in the manuscript were produced by Zhang J et al. (Int J Biol Macromol 107, 2018), which reported that mutation of the cleavage site (195-KKVK-198 to AAVA) severely affects the toxicity of the protein, showing that the proteolytic activation of Vip3Aa is linked to the insecticidal action. Moreover, experiments performed by different groups (Banyuls N et al. Sci Rep. 2018 and Shao E et al.

Toxins. 2020) have showed that mutation of residues T167 and S164, involved in the nucleation of the coiled-coil, displayed almost no toxic activity against cells of *S. frugiperda* and *S. litura*, respectively*.*

Page 5 paragraph 1; the sentence "Overall, the high-resolution structural features and the quality of the EM map have permitted unambiguous de novo atomic modelling of the protoxin and detailed structure and function analyses." is a little inaccurate. The authors should say that "Overall, the high-resolution structural features and the quality of the EM map have permitted unambiguous de novo atomic modelling of domains 1 - 3 of the protoxin" since not all of the pro-toxin was built using the map. And remove the comment about function analysis as only the structure was analysed (see comment about about testing the model using assays). Incorporation of some of the detailed structural analysis from Zheng paper would provide a more complete story.

We regret if this point was not accurate in the previous version of the manuscript. We are now reporting the structure of the complete protein in both states.

We have also removed the comment “functional analysis” as the reviewer requested. Moreover, we are providing a comparison with the Vip3B structure that overall is highly similar to the protoxin structure of Vip3Aa. This is especially true for domain I, II and III (RMSD value of -1 Å for 1932 residues) (**new Supplementary Fig. 6**), which is consistent with the elevated sequence similarity of this region (-70%). In relation to CBM domains, although sequence homology is lower (-40%), there is high structural similarity between the isolated domains but flexibility in the quaternary structure is observed as mentioned before. We have included the overall comparison between Vip3A and Vip3B protoxins in the result section of the revised manuscript.

Page 6 paragragh 2 line 6 "Domain III comprises residues 328-532 and contains three antiparallel 13-sheets that form a 13-prism fold strikingly similar to that found in members of the Cry insecticidal δ-endotoxins, and that has been shown to recognize the sugar moieties that decorate the receptors of the target cell". There are know beta-prism domains associated with other pore forming protein domains that are not demonstrated to bind to glycans (GNIP, Plu-MACPF). Take care not to imply glycan binding due to the existence of a beta-prism domain especially as there are no wet-lab experiments used to support this. I understand that it is implied not stated in the manuscript. Same comment for page 7 regarding domains IV and V. It is simply not known what they bind. In addition the crystal structure suggests a role or domain IV and V in ligand binding. And why is there the need for multiple binding sites?

We very much appreciate this comment. Numerous beta-prism domains, such as that of *Vibrio cholerae* cytolysin, are known to bind sugar moieties (Olson *et al*. J Mol Biol 2005; Levan *et al*. J Mol Biol 2013;). Although we simply intended to suggest that this might also be a possibility for Vip3, we acknowledge that it has not been demonstrated for any member of the family, and we indicate it now in the revised text.

Regarding domains IV and V, we agree with the reviewer that it is not known yet what type of glycan they may bind. However, a recent preprint shows that these domains have a strong preference for chitosan and chitin (Jiang K et al. Biorxiv. 2020). Moreover, according to the DALI server, these two domains share similarity with a glycoside hydrolase T*hermoanaerobacterium polysaccharolyticum* (TpolCBM16) (RMSD is -2 Å for -110 residues). TpolCBM16 is formed by two CBM domains in tandem separated by 23 residues (Bae B et al. J Biol Chem. 2008 283) and tandem CBM domains seem to increase affinity for polysaccharides (Boraston AB, Biochem J, 2004). Comparison between one TpolCBM16 with each domain IV and V shows that the glycan binding site is exposed to the solvent (see Figure below). Thus, we have acknowledged these observations in the new version of the manuscript.

**Domain IV**

**Figure. Comparison of CBM domains IV and V found in Vip3A with TpolCBM16.** Structure of TpolCBM16 (PDB: 2ZEY) is shown superposed to domain IV (in blue) and V (in purple) colored in pink over domain IV and in orange over domain V. The sugar cellopentaose found in TpolCBM16 structure is also shown.

**TpolCBM16 PDB (2ZEY)**

cellopentaose

**Domain V**

cellopentaose

**TpolCBM16 PDB (2ZEY)**

Do the structures provide insight into if there is a level of control of the timing of

protease activation? i.e. How is it prevented from pore formation prior to docking onto the receptor? How is pH regulation affected based on the structure?

We agree with the reviewer that this is an interesting question. Although it is currently well established that protease digestion is required for Vip3 function, is not clear when the activation occurs and whether there are other factors involved in the process. Our results show that, at least under certain conditions in vitro, the pore can be formed in the absence of the receptor and, therefore, it is possible that the conformational change could happen prior to receptor engagement. One possibility is that the receptor could act as an anchor to recruit activated toxins to the cell surface. However, we believe that the interaction of the receptor with the C-terminal domains could also stimulate further the transition to the active configuration, for instance by releasing the most N-terminal region of the protein that is clamped between the domain III and the core of the Vip3 in the protoxin state. We now include some to these observations in the revised discussion section.

As for the role of pH, toxin activation is often regulated by changes in the acidic conditions of the environment (Tc toxin, anthrax, etc). We have analyzed the trypsin treated Vip3Aa sample at different pH values (4, 7.5 and 10) using negative staining electron microscopy and, although we could identify both states, we could not detect significant changes in the protoxin/toxin ratio at different pHs (Figure below, panel a). Interestingly, however, there is an accumulation of negative charges on the apex of the Vip3 pyramid (helices 2 and 3), and on one side of the helices that flank the trypsin cleavage loop (helices 4 and 5) (Figure below, panel b), so we cannot rule out that a combination of changes in pH with other factors, such as interaction with a target receptor or the plasma membrane could have an effect on the activation process.

**Figure. Negative staining 2D averages and electrostatic surface. a**, Representative negative staining 2D averages of the trypsin digested sample. **b**, Electrostatic potential of the Vip3Aa protoxin.

There is comparison to Haemagglutinin (6 long helices?) and M2 class I (4 short helices) discussed. But how does the pore structure compare to the snap25/snare complex (4 long helices)?

We thank the reviewer for bringing to our attention the structure of the SNARE complex. We believe that the comparison with influenza's haemagglutinin and M2 protein are interesting because, in the first case they share several key steps during the activation reaction (i.e. proteolytic activation and release of an N-terminal hidden peptide that triggers a large conformational changes that leads to the formation of a coiled-coil), and the latter case illustrates how a 4-helix bundle can act as an ion channel. In addition, Vip3 also shares striking structural similarities with other tetrameric coiled-coils such as that formed by the Sendai virus P protein or, like the referee correctly points out, the SNARE complex, although they differ in their activation mechanism or mode of action. The hetero tetrameric SNARE complex is formed by a coiled-coil comprised by one helix of VAMP2, one helix of syntaxin1 and two helices of SNAP25. The coiled-coil (PDB: 5CCG; Zhou Q et al. Nature. 2015) is similar to the one observed in Vip3A showing a RMSD of 3.4Å for 232 residues (see Figure below). We now mention the similarity shared between these proteins in the manuscript.

**Figure. Superposition between the coiled-coil of Vip3A toxin with SNARE complex**. Domain I helices from toxin structure are colored by monomers (A in blue, B in pink, C in green and D in sand) and helices from SNARE complex are colored in yellow.

**Toxin	SNARE**

Minor:

Abstract: replace C-term with C-terminal

We have replaced it.

Supp fig 1b and supp fig 6b: higher resolution and larger images of the 2d class averages would be much appreciated

We have enlarged the images of the 2D averages in the **new Supplementary Fig. 1 and 7**.

Labelling the domains in fig 3c and b would be helpful to the reader.

We also believe that this would be useful. We have added a new panel (**new Fig. 3b**) to compare the domain organization of the toxin with the protoxin where domains are color-coded for clarity.

Reviewer #2 (Remarks to the Author):

This manuscript describes the architecture of protoxin and toxin forms of Vip3Aa obtained using Cryo-EM studies. The resolution obtained is sufficient to confirm previous structural predictions and when combined with modelling approaches gives near atomic resolution. By far the most interesting aspect of the described work is the large structural difference observed between the native and activated forms.

As the authors will be aware while this manuscript was being considered by Nature Communications an alternative Vip3 protoxin structure was published in Protein Science which was derived from a combination of X-ray crystallography and molecular replacement techniques. The two structures appear very similar but clearly the authors need to compare the two.

We agree with the reviewer that performing the comparative analysis is interesting and necessary. As mentioned to reviewer #1, we have included in the manuscript the comparison between the protoxin structures of Vip3A and Vip3B. The overall structural similarity is remarkably high, especially for domain I, II and III, which is consistent with their elevated sequence homology (-70% identity). Interestingly, although the newly resolved Vip3A CBM domains (IV and V) also share high structural similarity with their Vip3B counterparts (-40% sequence identity), they are found adopting different orientations. This observation could reflect that these CBM adopt different conformation in both isoforms, or that they require some degree of flexibility to engage their substrate. This analysis has been included in the revised text.

What is needed to make this a standout paper (not just another protein structure paper) and therefore suitable for the broad readership of Nature Communications is some in depth insight as to how the observed structural rearrangement associated with activation relates to the biological properties of the protein. Although the authors have attempted to do this, I find many of the explanations rather vague and needing more detail.

We apologize for being excessively concise at some points in our analyses. We have elaborated upon more of the ideas and descriptions in multiple parts of the manuscript, and we have included new supplemental figures and tables. Specific issues raised by the Reviewer are addressed below.

Particular areas that need further consideration are:

1) It is well established that these Vip3A toxins require activation for activity and this manuscript provides an explanation for that. The authors mention that multiple N-terminal cleavages take place including the removal of 11 amino acids at the very N-terminus as well as the cleavage at 198. The assumption is made that the 198 cleavage is the crucial one, which although consistent with the molecular shifts observed (eg Fig 4c), is not actually demonstrated. The authors should make specific reference to papers such as Zhang et al (<https://doi.org/10.1016/j.ijbiomac.2017.09.101)> who demonstrated that blocking this cleavage prevented activity. Ideally the authors should replicate this mutation and confirm that no structural rearrangement takes place.

We apologize to the reviewer if the discussion of the trypsin cleavage site was not clear. Although it has been shown that Vip3 can be digested in a number of sites, some of them have been demonstrated to occur only in vitro (Banyuls N et al. Int. J. Biol. Macromol. 2018). In particular, the role of the removal of the first 11 residues is unclear as they are predicted to be disordered and the first N-terminal amino acids do not seem to play an effect on the toxicity of the protein (Bhalla R et al. FEMS Microbiol Lett. 2005). In fact, since in our hands they also did not have any effect on the Vip3A activity, we deleted the first 9 amino acids from the construct to facilitate our initial crystallization trials and subsequent structural analysis.

However, several groups have shown that protease digestion generates two fragments of ~20 and 65 kDa that remain tightly associated, and that the primary cleavage site is located around residues 198-199 (Bel Y et al. Toxins. 2017; Chakroun M et al. Appl. Environ. Microbiol. 2014; Zack MD et al. Sci. Rep. 2017; Kunthic T et al. Biochim. Biophys. acta. Biomembr. 2017; Banyuls N et al. Int. J. Biol. Macromol. 2018). Moreover, like the reviewer correctly mentions, Zhang J et al. (Int J Biol Macromol, 2018) further pointed to the 195-KKVK-198 region as the main cleavage site, as mutations of these key residues hampered trypsin digestion and produced a drastic reduction in the toxic activity.

We agree with the reviewer that it would be interesting to characterize Zhang's trypsin loop mutant (195-KKVK-198 to AAVA) by EM. Therefore, we set up to investigate this variant but, unfortunately, we have tested over 65 clones and they all contained deletions or mutations that have prevented us from finding a

positive clone. However, beside the aforementioned previous biochemical reports, our structural data also points quite strongly to the 195-198 loop as the primary cleavage site. First, our results show that trypsin cleavage produces two fragments with molecular weights that are compatible with previous results and consistent with a single cleavage in the 195-198 loop (**new Supplementary Fig. 7a**). Moreover, our findings also show that trypsin digestion is required to trigger the activation of Vip3A, since in the protoxin state there is clear density for the protease cleavage loop in the EM reconstruction (**new Supplementary Fig. 5**), and we can only detect molecules with the coiled-coil needle after protease digestion (**new Supplementary Fig. 1 and 7**). As the reviewer requested, we have acknowledged the findings of Zhang et al and other groups and edited our discussion on this matter.

2) Palma et al’s paper on Vip3Ag is referenced (ref 22) but the authors should comment on the fact that using size exclusion chromatography Palma et al concluded that there were no significant structural differences between protoxin and toxin. This could just be a limitation of SEC.

As the reviewer comments, in the manuscript of Palma et al. (Toxins (Basel). 9, 165, 2017) SEC experiments were conducted with the protoxin Vip3A and trypsin-digested Vip3A and they did not observe a significant change in the elution for both unprocessed and processed protein. We have observed similar results and we believe that it could be a limitation of the resolution of the technique, since both samples consist of tetramers of identical molecular mass.

Moreover, we have calculated in silico de hydrodynamic volume of the protoxin and toxin using the program HullRad (Fleming, P.J. et al. Biophysical Journal, 114:856-869, February 27, 2018). For the toxin, we have extended the coiled-coil to account for the residues not traced due to the flexibility of EM map at the N-terminus. The translational and rotational hydrodynamic radius that accounts for a reliable measurement of the hydrodynamic volume shows that differences between structures are low, between 5-10Å, which explains the small difference in elution volumes and why it is so difficulty to separate these protein species by SEC.

| **HullRad** | **Protoxin** | **Toxin** |
| --- | --- | --- |
| Molecular Mass of the protein (g/mol) | 344125 | 341378 |
| Translational Hydrodynamic Radius (Å) | 73.48 | 78.35 |
| Rotational Hydrodynamic Radius (Å) | 76.08 | 86.10 |

3) On page 7 it is mentioned that 30% of the activated molecules maintained a protoxin configuration. An obvious explanation would be that 30% of the proteins were not activated. Although the authors claim to have used conditions in which

full activation was achieved I could not find any data (eg SDS gel) to confirm this. If full activation was achieved then I would like to see some discussion as to why such a high percentage of molecules did not “flip”. This also relates to point 4 below.

We regret if this point was unclear. We have included the SDS-PAGE gel with the result of the protease digestion in a new supplementary panel (**new Supplementary Fig. 7a**). The gel shows that trypsin cleavage goes to completion and generates two fragments of 20 and 65 kDa, which is consistent with a single cut in the 195-KKVK-198 loop and with previous reports (Bel Y et al. Toxins. 2017; Chakroun M et al. Appl. Environ. Microbiol. 2014; Zack MD et al. Sci. Rep. 2017; Kunthic T et al. Biochim. Biophys. acta. Biomembr. 2017; Banyuls N et al. Int. J. Biol. Macromol. 2018). Since the conformational change does not occur prior to trypsin treatment, our data suggest that protease digestion is required for activation. However, like the referee correctly points out, there is a percentage of molecules (~30%) that after cleavage do not switch conformations. We believe that this observation probably reflects the fact that trypsin digestion may not be the only factor involved in the activation of the toxin, and that other events, such as interaction with receptors, membrane, etc. could further stimulate the transition to the active configuration.

4) What the readership of this manuscript will really want to know is how this structural rearrangement reflects the biological activity of the protein. Although on page 3 is it noted that many other mechanisms other that pore-formation have been proposed, the rest of the manuscript seems to assume a pore-formation model. If I have misjudged this then I recommend that the authors also need to consider how the structure might relate to such other effects - including acting internally (<https://doi.org/10.1371/journal.ppat.1007347> ).

This matter has been subjected to numerous studies in the field. The majority of mechanisms proposed in the literature indicate that Vip3A can form pores and stable ion channels (Lee MK et al. Appl. Environ. Microbiol. 2003; Liu JG et al., J Invertebr Pathol. 2011; Kunthic T et al. *Biochim. Biophys. acta. Biomembr.* 2017), but other lethality pathways have been proposed. Although we cannot rule out any of these models, we believe that the dimension and length of the coiled-coil formed after trypsin digestion, which is more than sufficient to reach and permeate a lipid bilayer, and the similarity with other ion channels, support the pore formation mechanism. Interestingly, however, this mode of action can be consistent with other pathways. For instance, although it has been shown that Vip3 can recognize receptors located in the plasma membrane (Jiang K, Toxicon. 2016; Jiang K et al. Toxins. 2018), this would be also compatible with previous studies that show that Vip3 can be internalized (Jiang K et al. PLoS Pathog. 2018). This idea has also been suggested for other pore-forming systems such as the Tc toxins (Meusch D et al. Nature. 2014), where the pore

formation can be triggered directly on the plasma membrane or after endosomal internalization depending on the enviromental conditions. We have modified the manuscript to expand the discussion on the possible mechanisms of toxicity.

Assuming a pore-formation model though, then the model presented in Fig 5 requires further explanation. In this model structural rearrangement occurs following receptor binding yet in this manuscript it occurs (at least for 70% of the molecules) following protease treatment alone. The manuscript states that this process could be further stimulated by other factors like the interaction of with membrane receptors. This is not convincing – evidence indicates that receptor binding is not required for activation – not only in the in vitro studies in this manuscript but also in previous work where activated toxins could form pores in planar lipid bilayers. The authors need either to provide additional experiments to confirm that receptor binding does in fact further stimulate the activation process or they need to refine their Fig5 model. There are still many people working with Bt toxins who believe that receptors do not play a crucial role in activating the proteins – but simply act to concentrate them on the cell surface in the correct orientation and in sufficient numbers to have an effect.

We would like to thank the reviewer for pointing this up. Although the precise order or events are not fully understood, we agree that some aspects of the previous model figure could be misleading. Several studies have shown that Vip3 can permeate vesicles and lipid bilayers, and our data further supports the idea that the protein can be activated in the absence of any receptor. We did not intend to claim that the interaction with the receptor is required for activation, but simply imply that it could modulate the activity of the toxin -by stimulating the conformational change, concentrating, or targeting the protein to the appropriate site. We have modified **Fig. 5** in the revised manuscript.

5) Assuming once more a pore-formation model I feel that more clarity is required on what the nature of that pore will be. It is not clear to me whether the authors favour a model in which the helical bundle itself would allow ion movement which could then be followed by water via other means (eg aquaporins) to produce swelling/lysis, or whether the bundles would oligomerize to form a pore, or just whether the amphipathic helix 1 would oligomerize to form a pore. More discussion on these options is required

We apologize if we were not sufficiently clear. Domains III, IV and V form a steric shield that protrudes ~65 Å in all directions around the coiled-coil, which would prevent several tetramers from assembling into higher order oligomers to form a large pore in the membrane. Interestingly, however, it has been shown that ions and water molecules can be accommodated into the inner cavity of four-helix

bundles. We, therefore, consider that this is the most probable mode of action. In the case of activated Vip3 tetramer, the long parallel coiled-coil forms a strong dipole that could facilitate ion passage to the inner cavity. Moreover, we have identified cryo-EM density for a bound ion and several polar layers along the extension of the needle that further support this idea. Notably when we examined the dimensions and length of the coiled-coil, we noticed that the N-terminal ~45 residues, that contain the first alpha helix, appeared to be flexible. Since this region is highly amphipathic, it is tempting to speculate that this region could become ordered in contact with the membrane to form a coiled-coil with a cavity lined with hydrophilic residues. Importantly, however, these ideas are not antagonistic and, thus, we believe that the coiled-coil is crucial to perforate and permeate the lipid bilayer.

Finally there are some minor points which should be considered

i) On page 7 the structure prediction data can now be enhanced by the Zheng et al publication in Protein Science with respect to the carbohydrate binding domains

We agree with the reviewer. As mentioned to reviewer #1, we have improved the final structure adding domains IV and V. In particular, we used density subtraction and focused 3D classification, which has significantly improved the EM density of domains IV and V and has allowed us to build the three-dimensional structure of full-length Vip3A in both states.

ii) Page 9 are N@d layers real or a typo?

The N@d layers are the name given to heptads in coiled-coils that contain one hydrophilic residue in the *d* position of the heptad motif [I/V]xxNTxx which binds an ion (Hartmann MD et al, Proc Natl Acad Sci USA. 2009). In Vip3A, the coiled-coil contains the motif 160-VLINS-164 where N is in the *d* position (S can substitute the T) and binds a divalent metal ion that we have ascribed to magnesium.

iii) The last line of the manuscript is commonly thrown about – the authors should give some indication of how they believe that their data could be used to engineer proteins with increased activity. Which bits would be engineered and why?

Structural information can be extremely valuable to guide protein-engineering efforts. For this particular case, knowing the precise domain boundaries facilitates the design of chimeric proteins where different domains could be

**swapped to modify target specificity. This information can be used as well to increase the insecticidal toxicity, for example, by proving a more stable coiled-coil conformation or a more stable protoxin to avoid degradation for long-acting insecticidal activity. In summary, we believe that these structures will help to understand naturally occurring and human engineered mutants, and can be useful to produce new protein variants with enhanced biotechnological properties.**

**iv) Many of the references require italicization.**

**The references have been checked to match *Nat. Commun.* guidelines.**

**Reviewer #3 (Remarks to the Author):**

**Núñez-Ramírez et al. present the first structures of prepore (latent, protoxin) and pore (activated, toxin) states of the Vip3Aa system of the VIP3 family of proteins - an important system in agricultural science as a genetically encoded pesticide against Lepidopteran pests. Their study draws upon comparisons between two cryo-electron microscopy single particle reconstructions of the latent pro-toxin state with a trypsin-activated toxin (pore) form. Núñez-Ramírez et al. further provide a detailed analysis of the structural changes that occur between these forms and propose a model to describe the mechanism of these structural changes. This study provides a robust basis upon which to understand previously described mutations and effects of the Vip3Aa, thereby aiding the development of improved pesticidal proteins, as well as helping to shed light upon the mechanism/protein fold employed by this particular family. Overall the manuscript is well written and easy to understand, with a logical and fair presentation of the data.**

**We would like to thank the reviewer for the positive remarks.**

**Major comments:**

**1. The authors describe two extended loops in domain II (residues 221-226, 239­247) that supposedly stabilise the oligomer, how was this determined? Were any efforts made to assess the effects of these loops on the stability i.e. by truncating these to glycine or is this inferred solely from the structure? These loops are not clear in Fig. 1d.**

**The domains III, IV and V do not make any contact between the different Vip3 monomers. In contrast, domain II establishes numerous interactions with adjacent monomers and remains unchanged during the conformational changes that domain I undergoes to lead the activation of the protein. According to the**

PISA server, the interacting surface between adjacent monomers at the core, either in the protoxin or toxin structures is ∼1200 Å^2^. In particular, the vast majority of the interactions found in domain II (70%) lay in residues present in the loops 221-226 and 239-247 as it can be observed in Table 1. These two loops insert into the adjacent subunit and hold helix α4. Moreover, previous mutations performed by Banyuls et al (Sci Reports 2018) at loop 239-247 showed decreased of the insecticidal activity. The structural analysis shows that these loops act as a molecular staple that helps to stabilize the protein and, therefore, indicates that domain II plays a critical role in maintaining the three-dimensional organization of the tetramer.

**Table 1. Interactions between adjacent subunits A and D in the protoxin**. In yellow, are labelled the residues from domain II that belong to loop 221-226 and 239-247.

| **Subunit A** | |  | **Subunit D** | |  | **Distance (Å)** |
| --- | --- | --- | --- | --- | --- | --- |
| Gln | 147A | NE2 | Gly | 245D | O | 3.28 |
| Ser | 150A | CB | Phe | 244D | CD1 | 3.99 |
| Leu | 153A | O | Asn | 159D | OD1 | 2.68 |
| Asp | 154A | CA | Asn | 159D | CG | 3.86 |
| Ile | 156A | O | Asn | 159D | OD1 | 3.26 |
|  |  |  | Val | 160D | N | 3.47 |
| Val | 158A | N | Val | 158D | O | 3.27 |
| Asn | 163A | ND2 | Asn | 163D | ND2 | 3.21 |
| Leu | 166A | CD1 | Ser | 164D | CB | 3.90 |
| Thr | 167A | CA | Thr | 167D | CG2 | 3.66 |
| Thr | 170A | CG2 | Glu | 168D | CG | 3.73 |
| Tyr | 173A | CE2 | Leu | 243D | CA | 3.74 |
| Gln | 174A | C | Phe | 229D | CE1 | 3.89 |
| Gln | 174A | NE2 | Glu | 168D | O | 3.07 |
| Arg | 175A | NE | Asp | 225D | OD1 | 2.45 |
| Arg | 175A | NH2 | Tyr | 230D | OH | 3.06 |
| Arg | 175A | CG | Phe | 229D | CE1 | 3.53 |
| Lys | 177A | NZ | Asn | 241D | O | 3.05 |
| Tyr | 178A | CD2 | Phe | 229D | CD1 | 3.75 |
| Tyr | 178A | CE1 | Asn | 232D | CG | 3.76 |
| Val | 179A | CG2 | Val | 224D | CG1 | 3.52 |
| Glu | 181A | OE1 | Asn | 241D | ND2 | 3.23 |
| Lys | 182A | NZ | Glu | 228D | OE2 | 2.53 |
| Glu | 211A | CG | Ile | 301D | CG2 | 3.81 |
| Glu | 214A | O | Asn | 222D | OD1 | 3.38 |
| Glu | 214A | OE2 | Arg | 293D | NH2 | 2.73 |
| Ser | 218A | CB | Asn | 222D | C | 3.50 |
|  |  |  | Asp | 223D | CA | 3.69 |
| Lys | 221A | NZ | Asn | 222D | O | 2.80 |
| Asp | 225A | O | Asp | 225D | OD2 | 3.09 |

2. Figure 4b: “there is a clear peak of density coordinated by four symmetry related asparagine” - while the atomic model illustrates this, it would be helpful to guide readers if there were a map/model overlay that shows the quality of fit.

As the reviewer requested we have edited **Figure 4b** to overlay the EM density.

3. In fact, all maps shown in main figures alongside models appear to be the unsharpened maps. It is difficult to assess the agreement between the models and the EM density at the level of main chain. I would suggest a supplementary figure that shows map-to-model fit for different domains of the structure. A map-to-model Fourier Shell Correlation would also be advisable to demonstrate that over fitting has not occurred - this could be plotted on the same axis as Supp Fig 1c and 6c.

We agree that these figures are important to help to evaluate the goodness of the fit and the quality of the atomic model. We have added the map-to-model FSC curve and close up images in the **new Supplementary Fig. 1, 2, 7 and 8.**

4. Methods: The authors state that they use a spherical mask for the final

refinement, but I don't think they mean this, or at least it isn't clear to me. Usually one would use a mask generated from a low pass filtered map (this is why in sup fig 6 the red curve is present, to assess whether this mask introduces correlated information into the half sets i.e. interfering with the refinement). Surely, a low pass filtered protein mask was used, rather than a sphere (as one excludes more solvent). If a spherical mask was used for refinement, that's fine, but the resolution would underestimated and sharpening would likely create a lot of noise.

We regret if this point was unclear. For the three-dimensional refinements (auto-refine 3D in RELION) we tried both using a spherical and a tighter mask that followed the contour of the protein. These two approaches gave indistinguishable results in terms of resolution and interpretability of the map. Since the results were identical, we used the reconstruction obtained with the spherical mask, to make sure that we were not cutting any density, for the subsequent stages. However, the referee is correct, and for the post-processing step (i.e. resolution estimation and sharpening) a mask generated from a low-pass filtered map was always used to obtain an accurate resolution estimate and prevent the appearance of spurious noise. We have edited the method section to clarify this matter.

5. In two of the figures (sup fig 1f and sup fig 6f) the authors show maps that have regions that appear over sharpened (domains IV and V), generating noise. The core region is perfectly fine to model, but evidently domains IV and V at that sharpening value are not possible to model. Of course the authors acknowledge this - so that’s fine. Did the authors attempt to fit their homology models of

domain IV and V generated from Robetta into any of the maps (sharpened, unsharpened, filtered, etc.)? Were different sharpening values used for modelling or just one? Were the homology models refined against any maps (the unsharpened map, I note, still possesses distinct features that would enable fitting e.g. the connecting loop)? I suspect with a different sharpening value one could probably fit the homology models into the map presented.

We initially tried different sharpening strategies (global and local sharpening, with or without using the atomic model as input file) but, due to the presence of flexibility in this area, the quality of the map for the domain IV and V did not improve substantially, which only allowed us to roughly fit the unrefined domains in the unsharpened map. Importantly, however, like we have discussed with the reviewer #1, we have now improved significantly the density for the domains IV and V, which has allowed us to build and refine the complete structures of Vip3A in the protoxin and toxin states.

More precisely, we have used density subtraction to erase the core of the protoxin (domains I and II), and performed focused classification without alignments on the region that comprises domains III, IV and V. The results from the 3D classification showed that these three C-terminal domains move as a rigid body with respect to the core of Vip3 (**new Fig. 2c**). Due to their peripheral position, this movement has a more pronounced effect on domains IV and V, which explains why our initial model building efforts where not successful. We then repeated this process with the reconstruction of the activated toxin, which yielded very similar results.

To build domains IV and V, we generated a new homology model using Robetta (in this occasion the recently determined structure of Vip3B was used as a reference). We then performed several rounds of manual building and refining using the reconstruction from the 3D classification that showed the best connectivity. The refined models of domains IV and V were placed in all the monomers of the tetramers using the symmetry operators to generate the full-length structures.

6. Can the authors clarify in their methods for model building:

- What sharpening values were applied for model building? Did these differ from

those reported in table 1?

and

- Whether they built into one or more globally sharpened map(s), or,

- Whether they built into a locally filtered and globally sharpened map, or,

- Whether they built into a locally sharpened and locally filtered map.

We apologize if this matter was not clear. We have provided a detailed explanation on the methods used for model building in the previous point. Regarding the sharpening values, domains I, II and III of the toxin and protoxin where built and refined in their respective overall reconstructions (2.9 Å resolution). These maps were globally sharpened with the values reported in the table 1 (-64 and -69 Å^2^), which were automatically determined using RELION (Rosenthal PB and Henderson R. J Mol Biol. 2003).

Like we have discussed in the previous point, domains IV and V were built and refined in the best-resolved model from the focused 3D classification. Finally, after these domains were combined with the others to obtain the full-length structure, the complete tetramers were subjected to an ADP-only refinement cycle into the overall reconstructions (global sharpening values of -64 and -69 Å^2^) to allow the temperature factors of the domains IV and V to change slightly to better reflect the flexibility of the molecule.

7. If the authors only built into one map at one sharpening value, I would recommend reducing the sharpening that was used when considering domains IV and V, as this may allow further details and features to be observed. Alternatively some useful tools would be local_deblur in Scipion, relion_locres which produces a locally filtered map, or a locally sharpened map from phenix.autosharpen - these are useful when there is a large variation in local resolution. This may enable more confident fitting of domains IV and V.

We thank the referee for the suggestion. Like we have discussed before, we have tried to use different local sharpening procedures. However, we were able to obtain significantly better results using particle subtraction and focused classification that allowed us to build domains V and IV.

8. Lastly the authors have only deposit two EM maps. Personally, I feel the following maps should be uploaded. This benefits the community by enabling transparency and validation of the map, including assessment of model-to-map fit.

- The unsharpened map (1)

- The sharpened maps (1) used for building (whether this is globally sharpened, locally sharpened and/or locally/globally filtered - it doesn't matter, it's necessary to compare the models against). Multiple different sharpened maps or segmented maps may be uploaded e.g. focused refinements,

- The mask (1) used for the refinement/sharpening - to ensure the masks were not too tight.

- The half maps (2) enabling the FSC to be calculated i.e. to verify the resolution.

That would amount to five maps per structure. I would encourage the authors to consider depositing these.

Although we only reported two EMDB entries in the manuscript -one for the toxin and another for the protoxin- each one contained the respective unsharpened and globally sharpened map together with the FSC curve. However, we agree with the reviewer that it would be interesting to include additional information and we have now also deposited, as suggested, the mask used for resolution estimation and sharpening, the half maps, and the map obtained from the focused refinement of the C-terminal domains.

Minor comments:

- Can the authors provide an SDS-PAGE to show the purity of the final

recombinant Vip3a protein?

As the reviewer requests, we have included an SDS-PAGE gel in the **new Supplementary Fig. 7a** showing the purity of the protein before and after processing with trypsin.

- Abstract uses abbreviations “N-term” and “C-term” - use full terminology. We have replaced the abbreviations to the full terminology.

- Abstract “biotec” should be “biotech”?
It has been corrected.

- Figure 1 caption typo/unclear: “Inset displays the same orientation the EM map with the domains IV and V shown in grey”

Both the image and figure caption have been substantially modified.

- Methods: “[...] elongated negative staining particles reported [...]” should be “negative stained”

It has been corrected.

- Discussion: Suggest rephasing double negative “Not only domain I is not

eliminated by protease digestion [...]” to “Not only is domain I retained after protease digestion [...]”

As the reviewer suggest we have rephrased the sentence.

- Discussion - suggest rephrase: “[...] maintain fundamentally same contacts in the protoxin and toxin [...]” to “[...] maintain fundamentally the same contacts in the protoxin and toxin [...]”

We have corrected the typo.

- Discussion - suggest rephrase: “[...] F229, which locates at α6 [...]” to “[...] F229, which is located at α6 [...]”

It has been corrected.

- Supplemental figure 7 could be accompanied by a separate model superpositions of domains I, II and III in the pro- and toxin forms to help illustrate the conformational changes (or lack of) in each region of the complex.

We initially used the EM maps to show domains IV and V that were not modeled. Since we have now been able to resolve these domains, we have now replaced the EM density with a full-length model comparison between the toxin and protoxin monomers (**new Supplementary Fig. 9**).

- Figure 4g: “g” bold font

The typo has been corrected.

- Sup Fig 1b caption: “Workflow of image processing the protoxin particles.” rephrase “Workflow of image processing for the protoxin particles.”

The sentence has been changed.

- Sup Fig 2 caption: “Domains a highlighted in boxes colored as in Fig. 1a.” should be “Domains are highlighted in boxes colored as in Fig. 1a.”

The typo has been edited.

- Figure 1 and 3 should have scale bars (I note the scale bar on the 2D class average), however it is difficult to assess the dimensions of the complex.

We have added scales bars to the new Fig.1 and 3.

- Figure 2 caption typo/unclear:

a) Domain should not be plural: “domains III forms [...]” should be “domain III forms [...]”
b) “Groove” is misspelt.

The corresponding corrections have been introduced at Figure caption 2

**Reviewer comments, second version:**

Reviewer #1 (Remarks to the Author):

The authors have done a remarkable job of re-analysing the cryo-EM data in light of the published structures and in reference to other published concepts. It is really nice to see the "first in class" prepore to pore structures of Vip3A. This is exactly the information required for the field to understand the mechanism of action and I know that these two structures will become the launchpad for further investigation.

It is great that the authors implemented particle subtraction
and focused 3D classification to improve the EM structure interpretation. I think this really makes the paper a lot better. Deposited structures with all domains will be a far better (and citable) resource for other labs working on Vip3A and homologues.


Reviewer #2 (Remarks to the Author):

Unlike some of the other reviewers my expertise is not in structural biology so once again I shall restrict my review to the more functional aspects of the work.

In this revision the authors have satisfactorally addressed my previous comments with additional text and a modified figure 5. I feel now that the manuscript very nicely ties together their structure of Vip3Aa with previous research on the function of this protein. I only have some very minor suggestions for improvement:

1) Page 3 "recognizes then" should be "then recognizes"

2) Page 14 I feel that "the concentration of the toxin at the membrane" is better than "the concentration of the toxin in the membrane". Even though it is unclear what the nature of the pore is many other PFTs oligomerize prior to insertion.

3) My original comment on itacliztion in references still stands. I believe that Nature Communications still require species names to be italicized in references. Currently for example Bacillus thuringiensis is italicized in reference 2 but not in references 1,4,5,6 etc. Many other examples in other references (bacteria and insects).


Reviewer #3 (Remarks to the Author):

The authors have addressed all my previous comments and suggestions satisfactorily.

The addition of new focused classification experiments are an elegant solution to the problems created by the flexible regions of Vip3 and my concerns about interpretability of this region are resolved.

The changes made in both the figure legends and the methods section, including the updated figures, provide additional clarification and improve the overall readability. These will be helpful in reaching a broader audience.

Extremely minor comments about new additions to the text:

- Page 3: Suggest adverb ("then") come after the noun. "The activated protein then recognizes specific receptors"

- Page 6: Suggest "neighbouring" as an adjective, not the noun "neighbour".

- Page 6: Suggest "the exact role in Vip3A proteins has not yet been fully elucidated"

Kind regards,
Charles Bayly-Jones

**Author rebuttal, second version:**

Reviewer #1 (Remarks to the Author):

The authors have done a remarkable job of re-analysing the cryo-EM data in light of the published structures and in reference to other published concepts. It is really nice to see the "first in class" prepore to pore structures of Vip3A. This is exactly the information required for the field to understand the mechanism of action and I know that these two structures will become the launchpad for further investigation.

It is great that the authors implemented particle subtraction
and focused 3D classification to improve the EM structure interpretation. I think this really makes the paper a lot better. Deposited structures with all domains will be a far better (and citable) resource for other labs working on Vip3A and homologues.

We are grateful for the positive remarks.


Reviewer #2 (Remarks to the Author):

Unlike some of the other reviewers my expertise is not in structural biology so once again I shall restrict my review to the more functional aspects of the work.

In this revision the authors have satisfactorally addressed my previous comments with additional text and a modified figure 5. I feel now that the manuscript very nicely ties together their structure of Vip3Aa with previous research on the function of this protein. I only have some very minor suggestions for improvement:

1) Page 3 "recognizes then" should be "then recognizes"

We have corrected the word order in the revised manuscript.

2) Page 14 I feel that "the concentration of the toxin at the membrane" is better than "the concentration of the toxin in the membrane". Even though it is unclear what the nature of the pore is many other PFTs oligomerize prior to insertion.

The suggestion has been included in the text.

3) My original comment on itacliztion in references still stands. I believe that Nature Communications still require species names to be italicized in references. Currently for example Bacillus thuringiensis is italicized in reference 2 but not in references 1,4,5,6 etc. Many other examples in other references (bacteria and insects).

We thank the reviewer for pointing this out and we apologize for the misunderstanding. We have now italicized the species names in all the references.


Reviewer #3 (Remarks to the Author):

The authors have addressed all my previous comments and suggestions satisfactorily. 

The addition of new focused classification experiments are an elegant solution to the problems created by the flexible regions of Vip3 and my concerns about interpretability of this region are resolved.

The changes made in both the figure legends and the methods section, including the updated figures, provide additional clarification and improve the overall readability. These will be helpful in reaching a broader audience.

Extremely minor comments about new additions to the text:

- Page 3: Suggest adverb ("then") come after the noun. "The activated protein then recognizes specific receptors"

This issue has been corrected in the new version of the text.

- Page 6: Suggest "neighbouring" as an adjective, not the noun "neighbour".

We would like to thank the reviewer for the suggestion. We have replaced the noun with the adjective.

- Page 6: Suggest "the exact role in Vip3A proteins has not yet been fully elucidated"

The sentence has been changed in the manuscript.

**Reviewer comments, third version:**

**Author rebuttal, third version:**
